# Cognition of and Demand for Education and Teaching in Medical Statistics in China: A Systematic Review and Meta-Analysis

**DOI:** 10.1371/journal.pone.0128721

**Published:** 2015-06-08

**Authors:** Yazhou Wu, Liang Zhou, Gaoming Li, Dali Yi, Xiaojiao Wu, Xiaoyu Liu, Yanqi Zhang, Ling Liu, Dong Yi

**Affiliations:** Department of Health Statistics, College of Preventive Medicine, Third Military Medical University, Chongqing, 400038, China; Cardiff University, UNITED KINGDOM

## Abstract

**Background:**

Although a substantial number of studies focus on the teaching and application of medical statistics in China, few studies comprehensively evaluate the recognition of and demand for medical statistics. In addition, the results of these various studies differ and are insufficiently comprehensive and systematic.

**Objectives:**

This investigation aimed to evaluate the general cognition of and demand for medical statistics by undergraduates, graduates, and medical staff in China.

**Methods:**

We performed a comprehensive database search related to the cognition of and demand for medical statistics from January 2007 to July 2014 and conducted a meta-analysis of non-controlled studies with sub-group analysis for undergraduates, graduates, and medical staff.

**Results:**

There are substantial differences with respect to the cognition of theory in medical statistics among undergraduates (73.5%), graduates (60.7%), and medical staff (39.6%). The demand for theory in medical statistics is high among graduates (94.6%), undergraduates (86.1%), and medical staff (88.3%). Regarding specific statistical methods, the cognition of basic statistical methods is higher than of advanced statistical methods. The demand for certain advanced statistical methods, including (but not limited to) multiple analysis of variance (ANOVA), multiple linear regression, and logistic regression, is higher than that for basic statistical methods. The use rates of the Statistical Package for the Social Sciences (SPSS) software and statistical analysis software (SAS) are only 55% and 15%, respectively.

**Conclusion:**

The overall statistical competence of undergraduates, graduates, and medical staff is insufficient, and their ability to practically apply their statistical knowledge is limited, which constitutes an unsatisfactory state of affairs for medical statistics education. Because the demand for skills in this area is increasing, the need to reform medical statistics education in China has become urgent.

## Introduction

Medical statistics is an applied discipline that combines statistical principles and methods for data collection, collation, analysis, and inference with their applications in medical research [1–3]. Currently, knowledge of medical statistics is required for clinical medical students worldwide, particularly in China. Statistics is also a highly practical discipline that is an indispensable tool in many other fields of study. For medical undergraduates and graduates in Chinese colleges and universities, statistics is also a public and compulsory course. In the 21^st^ century, knowledge of medical statistics has become a required tool for clinicians and researchers who engage in clinical work and scientific research [4–7]. However, the ability of clinicians and medical students to apply their knowledge of medical statistics is unsatisfactory. Because of an inadequate grasp of medical statistics, these students lack the skills necessary for the application of statistical design and analysis. Consequently, they often misuse statistical methods. This insufficiency results in the failure of their research papers to be accepted and published by journals because the papers do not meet scientific standards, which results in a waste of valuable, specialized resources [8–9]. Therefore, the evaluation of the cognition of and demand for medical statistics among medical staff and medical students in a manner that reflects the developing trends in biological medicine and conveys the necessity of a comprehensive understanding of medical statistics has become an urgent issue that must be addressed [10–12].

At present, although many publications have focused on the teaching and application of medical statistics in China, few studies comprehensively evaluate the cognition of and demand for medical statistics. In addition, among such studies, results differ and are insufficiently comprehensive and systematic. Fortunately, these publications contain strongly representative and relatively complete data. Therefore, we performed a comprehensive search of the relevant literature on the cognition of and demand for medical statistics. Using comprehensive comparisons of the cognition of and demand for medical statistics among undergraduates, graduates, and medical staff, we conducted a comprehensive review and evaluated the current status of the cognition of and demand for medical statistics. Finally, we analyzed and summarized current challenges with respect to medical statistics and proposed a targeted improvement strategy. This strategy can serve as a reference and basis for innovative education and teaching reform in medical statistics courses and improves the ability of clinical researchers and medical students to use analytical statistical knowledge to solve practical problems in medicine.

## Materials and Methods

### Inclusion and Exclusion Criteria

The literature inclusion criteria were as follows:

1. All of the included studies were investigations.
2. The subjects of this study included three types of individual found at the medical university and hospital: undergraduates, graduates, and medical staff.
3. The study investigated the cognition of and demand for medical statistics methods and the conditions for using statistics software among undergraduates, graduates, and medical staff in China.
4. End indicators included the cognition level, the demand level, and the usage level for medical statistics methods or related software.

The literature exclusion criteria were as follows:

1. Literature with non-exploitable data or a vague concept of cognition and demand in the study.
2. Review literature.
3. Literature with serious data omissions.

### Literature Search and Selection

We used “teaching methods” as the title word and “cognition”, “demand” or “need”, “medical statistics” or “health statistics”, and “biostatistics” or “biometry” as keywords to search the following databases: PubMed, Medline, Chinese Biomedical Literature Database, China Doctoral Dissertations Full-text Database, Chinese Scientific and Technological Journals Database, Traditional Chinese Medicine Database, China Doctoral Dissertations Full-text Database, China Masters’ Theses Full-text Database, and CENTRAL from the Cochrane Library for the period January 2007 to July 2014. Additionally, we manually searched for relevant information stored at the Third Military Medical University’s library. The period specified in these searches ranged from the creation date of each database to July 2014. The format used for the Medline search was as follows:

#1 teaching methods
#2 cognition
#3 demand or need
#4 medical statistics or health statistics or biostatistics or biometry
#5 #1 and #2 and #3 and #4

### Quality Assessment

The included observational studies were subjected to a comprehensive quality assessment using the Newcastle-Ottawa Scale (cross-sectional/prevalence study) as a guide [13]. This quality evaluation was performed in a blind manner by two researchers (DY and LL) from the second research team, who assigned quality scores to the included studies. The studies that were awarded different quality scores by the two researchers were referred to a third researcher (YZ) from this research team for evaluation, and a final quality score was obtained (S1 Table).

### Data Extraction

Three investigators participated in the data extraction for all of the publications included in the study. Information regarding the first author, publication year, total number of cases included in the study, study object, and endpoint evaluation indicators (i.e., the measurement data and the enumeration data) was extracted. First, one investigator (GL) performed the data extraction. Then, the second investigator (LZ) re-examined the publication and verified the results. Differences were discussed with the third investigator (YW), and consensus was reached by discussion.

In terms of data extraction and quantification, the cognition rate, the demand rate, and the usage rate were used as end indicators. Cognition refers to the ability to know, understand, or master a statistical method and apply this method to solve problems. The cognition rate refers to the percentage of individuals who possess this ability among the surveyed population. Demand refers to the degree of demand of a statistical method in solving problems, and the demand rate is the percentage of the individuals who must use this method to solve problems among the surveyed population. The usage rate is the percentage of the individuals who use statistical software among the surveyed population.

### Statistical Analysis

EpiData 3.1 (The EpiData Association, Odense, Denmark) and Excel were used for data entry and collation. SPSS 18.0 (SPSS, Inc., Chicago, USA) was used for the statistical processing and analysis of the data. Stata 11.0 (Stata Corp LP, USA) was used to analyze the collected research data for meta-analysis. The extracted dichotomous data and multi-category data were uniformly converted to dichotomous variables, which were then expressed as a percentage or constituent ratio and rate. The endpoint data were evaluated using the pooled rate and 95% confidence interval (CI) based on the levels of cognition and demand for theory courses and software in medical statistics. For example, in the survey that targeted the cognition of the t-test, there were three choices “proficient use” (n_1_), “general use” (n_2_), and non-use (n_3_). The positive data on “proficient use” (n_1_) and “general use” (n_2_) were pooled for analysis, and the cognition rate of the t-test was obtained as follows: (n_1_+n_2_) / (n_1_+n_2_+n_3_). Subgroup meta-analysis for undergraduates, graduates, and medical staff was also used. If the heterogeneity across the studies was within the acceptable range (I^2^<50%), a fixed-effects model was used to combine the studies. Otherwise, a random-effects model was used. P<0.05 indicated a statistically significant difference.

## Results

### Demographic Characteristics of the Studies

**Fig 1:**
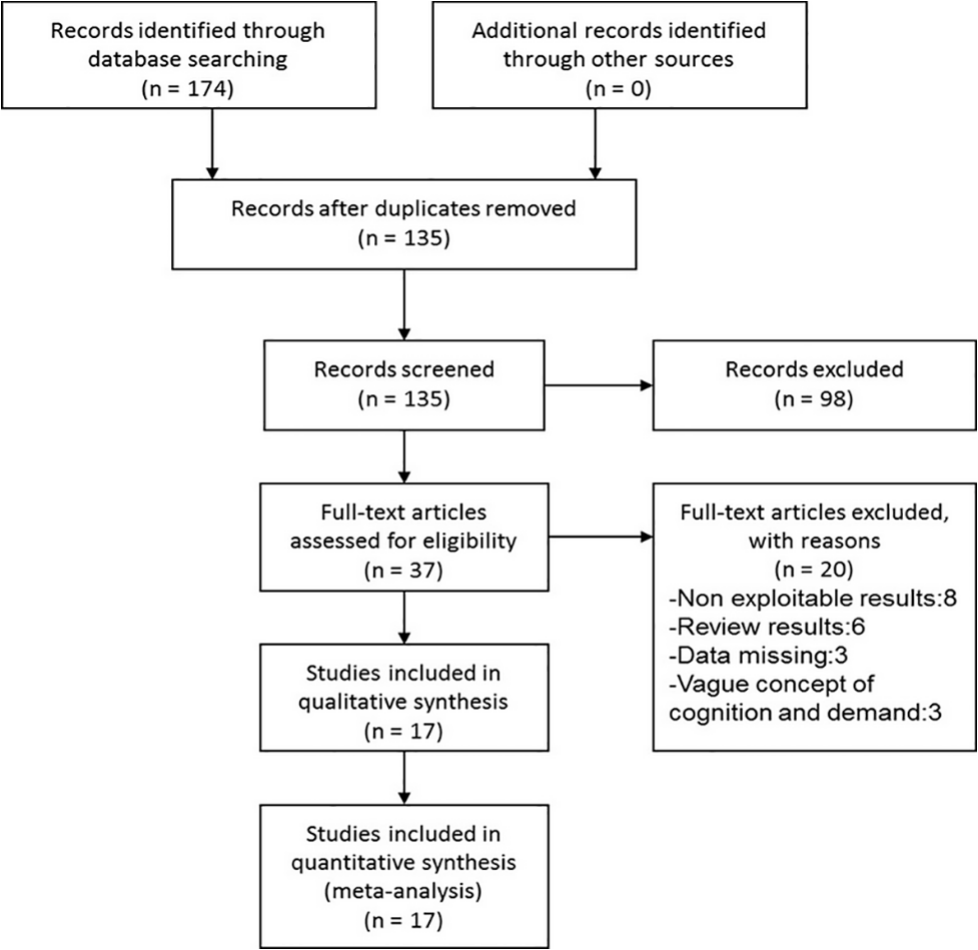
Flowchart of the Included Studies.

In total, 174 research articles on the cognition of and demand for medical statistics were identified by searching electronic databases and other sources. Based on the inclusion and exclusion criteria, 98 articles, including duplicate publications, articles with mismatched titles, and articles with mismatched subjects, were excluded. The remaining 37 articles were thoroughly reviewed, and the following 20 studies were excluded: eight articles with non-exploitable results, six review articles, three articles with missing data, and three articles with vague concepts of cognition and demand. Thus, 17 studies were included in this study for the systematic review [14–30]. Fig 1 displays a flowchart of the included studies.

Table 1 presents the basic demographic characteristics of the undergraduates, graduates, and medical staff in the included studies. The undergraduates included the combined Bachelor of Science/Doctor of Medicine (BS/MD) Program (i.e., 7- or 8-year) students. The graduates also included doctor of philosophy (PhD) students. The medical staff also included clinicians, nursing personnel, and health-service management personnel. In China’s education system, undergraduates are college students who are pursuing a bachelor’s degree. Graduates are students who are pursuing a master’s degree after obtaining a bachelor’s degree. PhD students are pursuing a doctoral degree after obtaining a master’s degree. Clinicians and nursing personnel are health-care workers who are employed at a hospital.

**Table 1:** Basic Demographic Characteristics of the Included Studies.

Author	Publication year	Sample	Object of study	Theory Course		Software		
				Cognition (n)	Demand (n)	Cognition (n)	Demand (n)	Use (n)
Jing Wang[14]	2007	197	Undergraduate	N/A	N/A	N/A	N/A	⒄
Yalin Sun[15]	2009	20	Combined BS/MD program (7-year) students	⑴⑵⑶⑷⑸⑹⑺⑻⑼⑽⑾⑿⒀⒆	⒆	⒇	⒇	N/A
Xiuqiang Ma[16]	2009	149	PhD students	⑴⑵⑶⑷⑸⑹⑺⑼⑽⑾⑿⒀⒁⒂⒃	⑴⑵⑶⑷⑸⑹⑺⑼⑽⑾⑿⒀⒁⒂⒃	⒇	⒇	⒄⒅
Hong Meng[17]	2009	211	PhD students	⑴⑵⑶⑷⑸⑹⑺⑼⑽⑾⑿⒀⒁⒂⒃⒆	⑴⑵⑶⑷⑸⑹⑺⑼⑽⑾⑿⒀⒁⒂⒃⒆	N/A	N/A	⒄⒅
Yugui Fang[18]	2011	776	Nursing personnel	⒆	⑺⑻⒆	⒇	⒇	N/A
Canqing Yu[19]	2011	249	Graduate	N/A	N/A	N/A	37	⒄⒅
Guangzi Qi[20]	2011	216	Graduate	⑴⑵⑶⑷⑸⑹⑺⑼⑽⑾⑿⒀⒁⒂⒃⒆	⑴⑵⑶⑷⑸⑹⑺⑼⑽⑾⑿⒀⒁⒂⒃⒆	⒇	⒇	N/A
Juan Tang[21]	2011	200	Clinician	⑴⑵⑶⑷⑸⑹⑺⒆	⒆	⒇	⒇	
Dongmei Hu[22]	2011	95	Graduate	⑴⑵⑷⑸⑹⑺⑻⑼⑿⒆	⒆	N/A	N/A	N/A
Huayan Zhang[23]	2011	50	Medical staff	⒆	⒆	N/A	N/A	N/A
Haiyan Ma[24]	2011	104	Health service management	⒆	⒆	⒇	⒇	N/A
Juan Wu[25]	2011	142	Undergraduate	⒆	N/A	N/A	⒇	N/A
Yanqi Zhang[26]	2012	74	Combined BS/MD program (8-year) students	⑴⑵⑶⑷⑸⑹⑺⑻⒆	⑻⒆	⒇	N/A	⒄
Yanfang Zhao[27]	2013	473	Graduate	⑴⑵⑶⑷⑸⑹⑺⑼⑽⑾⑿⒀⒁⒂⒃⒆	N/A	N/A	⒇	⒄⒅
Yan Zhu[28]	2013	859	Undergraduate	⑴⑹⒆	⑻	⒇	⒇	N/A
LiXia Li[29]	2013	117	Undergraduate	⒆	⒆	N/A	N/A	N/A
Yazhou Wu[30]	2014	163	Graduate	⑴⑵⑶⑷⑸⑹⑺⑻⑼⑽⑾⑿⒀⒁⒂⒃⒆	⒆	⒇	N/A	N/A
Yazhou Wu[30]	2014	285	Undergraduate	⑴⑵⑶⑷⑸⑹⑺⑻⒆	⒆	⒇	N/A	N/A

**Note:** n: The sample sizes for cognition, demand, and use. N/A: not applicable. (1). Descriptive statistics, (2). t-test, (3). ANOVA, (4). Chi-squared test, (5). Nonparametric test, (6). Correlation and regression, (7). Statistical graphs and tables, (8). Statistical design, (9). Multiple ANOVA, (10). Analysis of covariance, (11). Multiple linear regression, (12). Logistic regression, (13). Survival analysis, (14). Discriminant analysis, (15). Clustering analysis, (16). Principal components analysis and Factor analysis (PCA & FA),(17). SPSS, (18). SAS, (19). Overall cognition of and demand for medical statistics, (20). Overall cognition of and demand for software.

The study protocol was approved by the Third Military Medical University Ethics Committee, and informed consent was obtained from all of the participants. This study complied with the Helsinki Declaration.

### Quality of the Included Studies

In the Newcastle-Ottawa Scale (cross-sectional/prevalence study; NOS) results, 70.58% (i.e., 12 of 17) of the included studies achieved more than four NOS-item scores, 23.52% (i.e., 4 of 17) of the included studies achieved more than seven NOS-item scores, and 5.90% (i.e., 1 of 17) of the included studies achieved more than NOS-item scores (S1 Table).

### Overall Cognition of and Demand for Medical Statistics Competency Issues

**Fig 2:**
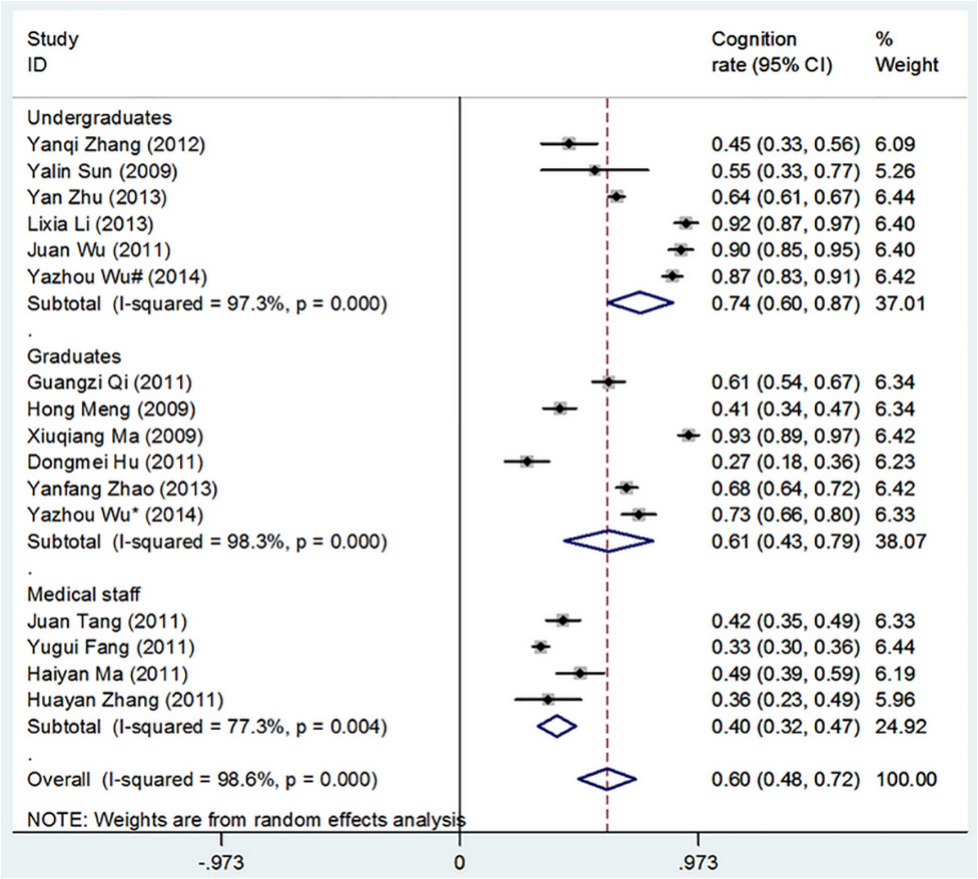
Overall Cognition of Theory in Medical Statistics Courses. (I-squared and *P* were the heterogeneity test criteria; ◇pooled cognition rate;—■—, cognition rate and 95% confidence interval).

**Fig 3:**
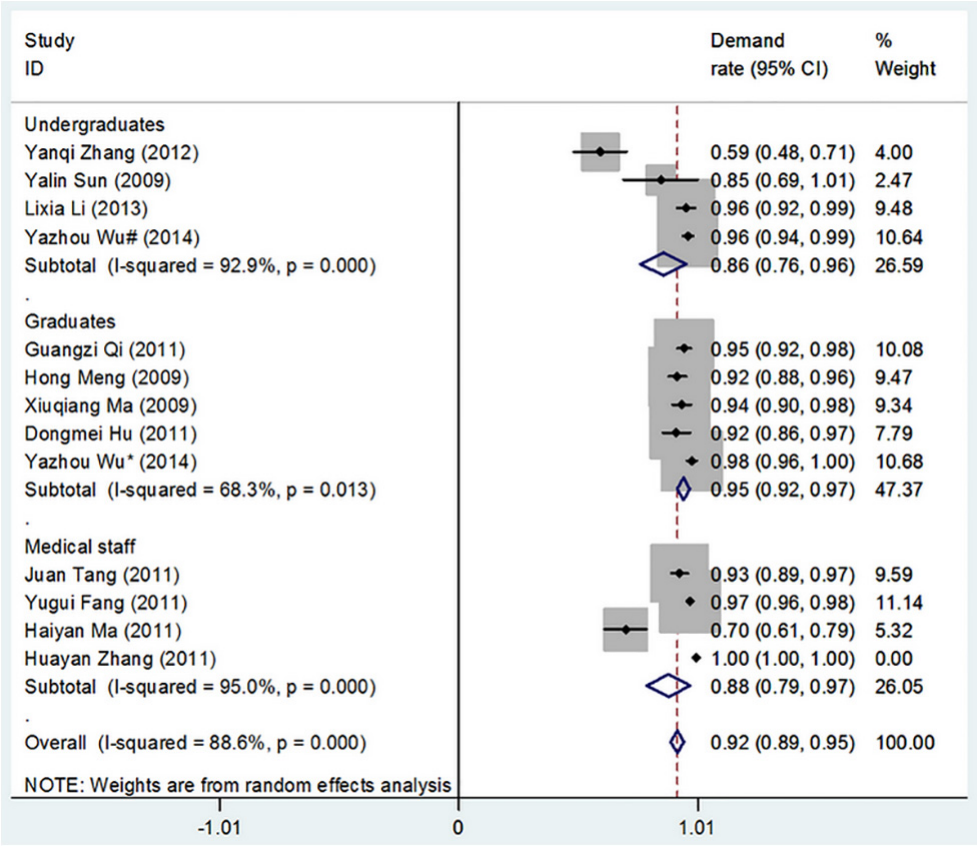
Overall Demand for Theory in Medical Statistics Courses. (I-squared and *P* were the heterogeneity test criteria; ◇pooled demand rate;—■—, demand rate and 95% confidence interval).

**Fig 4:**
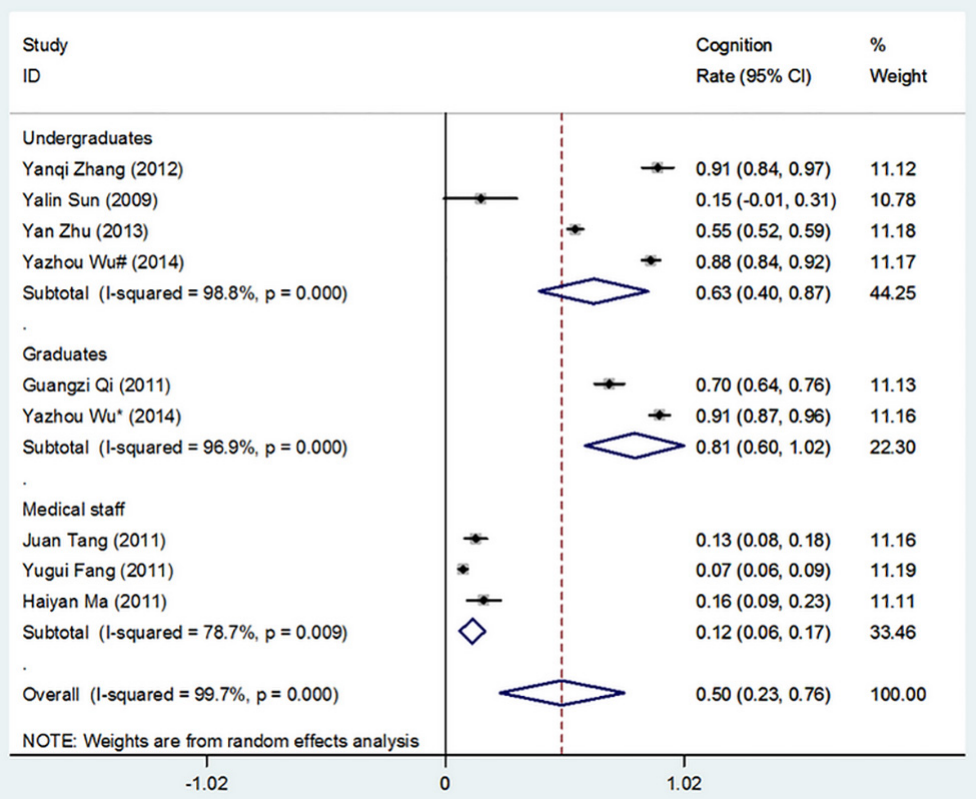
Overall Cognition of Statistical Software. (I-squared and *P* were the heterogeneity test criteria; ◇pooled cognition rate;—■—, cognition rate and 95% confidence interval).

**Fig 5:**
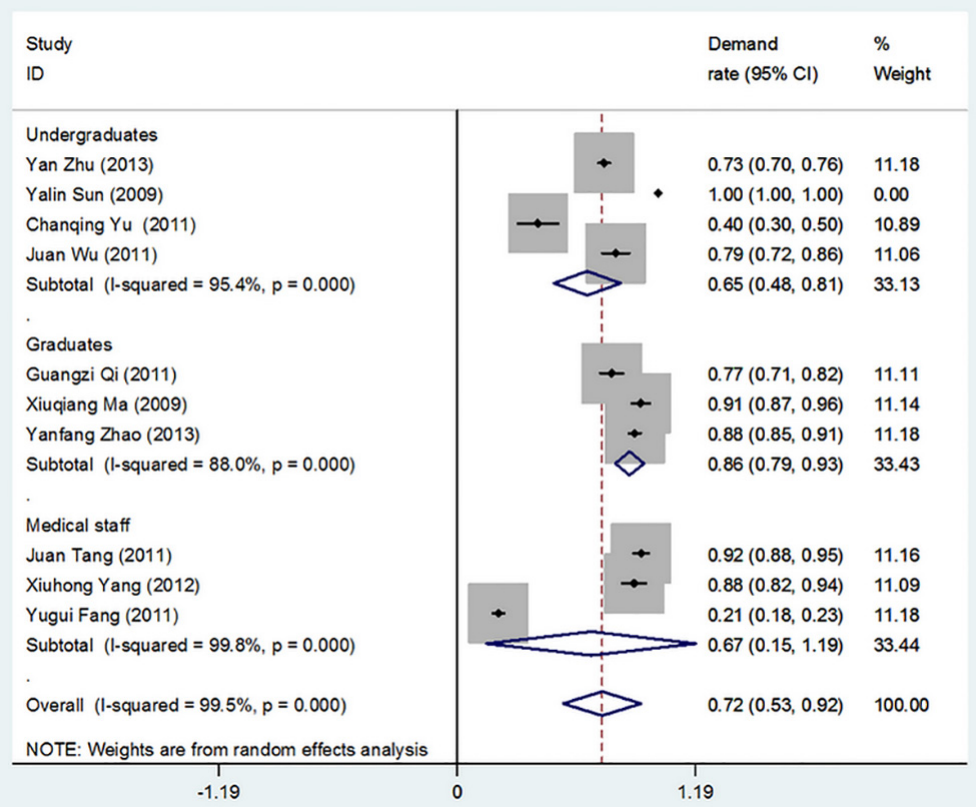
Overall Demand for Statistical Software. (I-squared and *P* were the heterogeneity test criteria; ◇pooled demand rate;—■—, demand rate and 95% confidence interval).

Table 2 shows the overall cognition of and demand for theory and software with respect to medical statistics competency issues among undergraduates, graduates, and medical staff. Figs 2–5 show the merged results of the meta-analysis of the overall cognition of and demand for medical statistics theory and software. The results from Table 2 and the figures also reveal the following: The cognition rates for medical statistics theory in undergraduates, graduates, and medical staffs were 73.5%, 60.7%, and 39.6%, respectively, and the cognition rates for statistics software were 63.3%, 80.8%, and 11.5%, respectively. The demand rates for medical statistics theory among undergraduates, graduates, and medical staff were 86.1%, 94.6%, and 88.3%, respectively, and the demand rates for statistics software were 64.7%, 85.7%, and 66.7%, respectively.

**Table 2:** Overall Cognition and Demand for Medical Statistics Theory and Software.

	Objects	Cognition				Demand			
		Sample	Pooled rate (%)	95% CI (%)	*P*	Sample	Pooled rate (%)	95% CI (%)	*P*
**Theory**	Undergraduates^#^	1,497	73.5	60.2–86.8	0.000*	496	86.1	76.4–95.8	0.000*
	Graduates^◇^	1,307	60.7	42.7–78.7	0.000*	834	94.6	91.9–97.2	0.000*
	Medical staff^◆^	1,130	39.6	31.9–47.3	0.000*	1,130	88.3	79.1–97.4	0.000*
**Software**	Undergraduates^#^	1,238	63.3	40.0–86.5	0.000*	1,113	64.7	48.1–81.2	0.000*
	Graduates^◇^	379	80.8	59.7–100.0	0.000*	834	85.7	78.6–92.8	0.000*
	Medical staff^◆^	1,080	11.5	6.1–16.9	0.000*	1,092	66.7	52.7–91.5	0.012*

*Statistical significance

# Includes undergraduates, combined BS/MD program (8-year) students, and combined BS/MD program (7-year) students.

◇ Includes graduates and PhD students.

◆ Includes clinicians, medical staff, nursing personnel, and health service management personnel.

### Cognition of and Demand for Statistical Methods and Software

**Fig 6:**
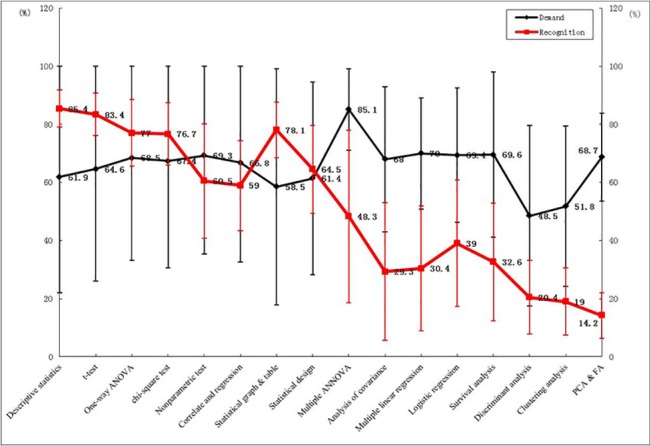
Relative Rate of Change in the Cognition of and Demand for Basic and Advanced Statistical Methods. (Basic statistical methods: descriptive statistics, t-test, one-way ANOVA, chi-squared test, nonparametric test, correlation and regression, statistical graphs and tables as well as statistical design. Advanced statistical methods: multiple ANOVA, analysis of covariance, multiple linear regression, logistic regression, survival analysis, discriminant analysis, clustering analysis, and PCA & FA.).

Table 3 presents the meta-analysis of the statistical methods and software with respect to medical statistics competency issues. (The meta-analysis results of each method are shown in S1–S10 Figs) Fig 6 shows the relative rates of change in the cognition of and demand for statistical methods.

**Table 3:** Meta-analysis of Statistical Methods and Software in Medical Statistics.

Statistical method and software		Cognition				Demand			
		Sample	Pooled rate (%)	95% CI (%)	*P*	Sample	Pooled rate (%)	95% CI (%)	*P*
**Basic statistical methods**	Descriptive statistics	2,745	85.4	79.0–91.8	0.000*	576	61.9	22.0–100.0	0.002*
	t-test	1,886	83.4	76.1–90.7	0.000*	576	64.6	26.0–100.0	0.001*
	One-way ANOVA	1,791	77.0	65.5–88.5	0.000*	576	68.5	33.2–100	0.000*
	Chi-squared test	1,886	76.7	66.0–87.4	0.000*	576	67.4	30.6–100	0.000*
	Nonparametric test	1,886	60.5	40.8–80.2	0.000*	576	69.3	35.4–100	0.000*
	Correlation and regression	2,745	59.0	43.4–74.4	0.000*	576	66.8	32.6–100	0.000*
	Statistical graph & table	1,886	78.1	68.5–87.7	0.000*	1,352	58.5	17.8–99.1	0.005*
	Statistical design	637	64.5	49.3–79.7	0.000*	1,709	61.4	28.2–94.6	0.000*
**Advanced statistical methods**	Multiple ANOVA	1,327	48.3	18.6–77.9	0.001*	576	85.1	71.1–99.2	0.000*
	Analysis of covariance	1,232	29.3	5.6–53.1	0.015*	576	68.0	43.0–92,9	0.000*
	Multiple linear regression	1,232	30.4	8.8–51.9	0.006*	576	70.0	50.9–89.2	0.000*
	Logistic regression	1,327	39.0	17.2–60.9	0.000*	576	69.4	46.3–92.5	0.000*
	Survival analysis	1,327	32.6	12.4–52.9	0.002*	576	69.6	41.2–98.0	0.000*
	Discriminant analysis	1,212	20.4	7.8–33.1	0.002*	576	48.5	17.4–79.7	0.002*
	Clustering analysis	1,212	19.0	7.5–30.5	0.001*	576	51.8	24.2–79.5	0.000*
	PCA & FA	1,212	14.2	6.3–22.1	0.000*	576	68.7	53.6–83.9	0.000*
**Statistical software**		**Sample**		**Rate** ^**△**^ **(%)**		**95% CI (%)**		***P***	
	SPSS	1,353		55.0		21.4–88.7		0.001*	
	SAS	1,082		15.0		1.5–28.5		0.029*	

*Statistical significance

△the usage rate.

#### Cognition of Statistical Methods

Among the basic statistical methods, the highest cognition was for descriptive statistics (85.4%), followed by the t-test (83.4%) and one-way ANOVA (77%). The lowest cognition was for correlation and regression (59%) and the nonparametric test (60.5%), and the cognition for experimental and survey design was only 64.5%. Among the advanced statistical methods, the highest cognition was for multiple ANOVA (48.3%), followed by logistic regression (39%) and survival analysis (32.6%). The lowest cognition was for principal component analysis and factor analysis (PCA & FA) (14.2%).

#### Demand for Statistical Methods

Among the basic statistical methods, the highest demand was for the nonparametric test (69.3%), followed by one-way ANOVA (68.5%) and the chi-square test (67.4%). The lowest demand was for statistical graphs and tables (58.5%), and the demand for experimental and survey design reached up to 61.4%. Among the advanced statistical methods, the highest demand was for multiple ANOVA (85.1%), followed by multiple linear regression (70%) and survival analysis (69.6%). The lowest demand, which was for discriminant analysis, reached up to 48.5%.

#### Use of Statistical Software

The usage rates for the SPSS software and SAS were only 55% and 15%, respectively.

## Discussion

### Problems Discovered

Table 2 reveals that there are substantial differences with respect to the cognition of medical statistics theory and software among undergraduates, graduates, and medical staff. In particular, medical staff exhibit less cognitive ability. For example, the surveys reported by Tang Juan and others indicate that 95.5% of clinicians cannot use more complex, advanced statistical methods, such as multiple linear regression and survival analysis, and only 13.0% of clinicians are familiar with SPSS or software or SAS [22]. Table 2 also shows that graduate students have more demand for medical statistics theory and software than undergraduates and medical staff. Such results indicate that it is critical for graduates to master and apply statistics during their graduate training. Ma’s research also reveals that the vast majority of PhD students realize that medical statistics is highly important for medical scientific research and believe that they should learn some course-related statistics during the doctoral stage [18]. Wang et al. consider it to be crucial for graduate students to correctly apply statistical methods to effectively conduct scientific research and to improve the quality of their medical degree theses [31].

Table 3 and Fig 6 show that the cognition of basic statistical methods is higher than that of advanced statistical methods. In contrast, the demand for certain advanced statistical methods, such as multiple ANOVA, multiple linear regression, and logistic regression, is higher than for basic statistical methods. However, the survey results also reveal certain differences because the study subjects were different. For example, the survey by Meng et al. indicates that 50% of PhD students believe they only need a simple review of statistics knowledge, whereas 63.5% of PhD students believe that advanced statistical methods should be explained in detail [19]. In the survey by Qi et al., 80% of graduate students believe that basic statistical methods should be explained in detail. However, fewer than 55% of graduates express a demand for advanced statistical methods [20].

These results suggest that among undergraduates, graduates, and medical staff with different education levels, many understood of and could apply commonly used, basic statistical methods. However, their overall ability to practically apply their statistical knowledge was insufficient, which was primarily apparent in their overall insufficient application of statistical design and advanced statistical methods, their lack of familiarity with basic statistical methods, and their lack of critical thinking and software application in the scientific context. Chen and Hu et al. believe that compared with other medical courses, medical statistics includes abstract concepts, numerous formulas, and a strong logic component, which are closely related to mathematics, and the course is generally considered to be more difficult to study by Chinese students [32]. Hu et al.’s survey results reveal that teachers do not focus on the memorization and derivation of calculus formulas and that they should emphasize the application of statistical methods and the operation of statistical software in future lectures [14]. In brief, more than 90% of medical staff members and medical students recognized the practical importance of medical statistics and the necessity of offering a medical statistics course. However, their understanding and mastery of statistical methods, cognition of statistical software, and capacity for research design were deemed unsatisfactory.

These results also suggest the following: The demand of medical students, particularly graduates, for an increased knowledge of medical statistics was manifested in their desire to increase the course offerings and quality of teaching in research design, advanced statistical methods, and statistical software application. The demands of medical staff centered on improving their ability to solve practical clinical problems, and a majority of subjects wished to gain statistical knowledge through continuing education.

### Adopting Strategies

Although this study is a comprehensive evaluation of various investigations, based on the conditions existing at the time of this study and the demand for medical statistics among medical students and medical staff, the following concrete strategies to improve medical statistics teaching and education in China are proposed:

#### First, attention should be paid to the cultivation of statistical thinking

During the process of studying and applying medical statistics, one should convert practical problems into statistical problems. It is particularly important to employ statistical thought processes. However, the cultivation of statistical thinking cannot be separated from statistical applications [33–36]. Only when statistical thought processes and applications have been used to solve practical problems can we intensify, solidify, and improve statistical thinking. Therefore, medical statistics courses should increase their focus on practicality and case analyses to improve student abilities to rigorously apply statistical methods and scientific thinking [14, 26].

#### Second, content regarding statistical design and advanced statistical methods should be improved

The percentage of medical students who wish to study experimental design and to gain familiarity with advanced statistical methods is relatively high, which suggests that these students are no longer satisfied with only using simple statistical methods to process data. Therefore, we can use flawed cases in research design to explain the principles of statistical design and thereby improve the research design skills of medical students (particularly master’s degree students) [22, 27, 37–39]. We should also combine medical cases and select commonly used advanced statistical analyses for medical research to teach the students, expand their horizons, and enhance their understanding of and ability to apply these difficult statistical methods [17, 40–42].

#### Again, the ability to use statistical software and to apply statistics to practical and clinical applications should be enhanced

Medical statistics education not only concerns teaching statistical theories and methods but also training students to apply these theories to solve practical problems. Therefore, we should strengthen statistical software training and incorporate a large variety of case studies and statistical software demonstrations to emphasize the real-life conditions in which statistical methods and thinking processes are applied [19, 23, 43]. This approach will enhance medical student competency regarding the use of statistical software to process and explain statistical results [43, 44].

#### Finally, we should attempt to make the teaching methods flexible and the teaching styles diverse

For medical students, the teaching process should focus on the student’s subjective initiative. In addition, diversified teaching methods should be developed. Case-based learning (CBL) [33, 45–46] and problem-based learning (PBL) should be applied [47–48] to provide opportunities for interactive interest in the coursework [19, 49]. For medical staff, various continuing education strategies should be considered, such as guest lectures or short-term training sessions [14, 17, 24].

### Study Limitations

First, although the sampled research subjects were relatively representative (i.e., clinicians, PhD students, graduates, combined BS/MD program (7- or 8-year) students, and undergraduates), these subjects were studied at a single institute. In addition, the number of surveyed samples was not sufficiently large or representative of the actual population. However, the surveyed students and personnel were natives of many and various regions of China, which is a strength of this study. Moreover, the questionnaire results in the original studies might be subjective, and a certain amount of exaggeration may occur if a respondent does not wish to appear ignorant.

The distribution was relatively broad, and the assorted institutes (or centers) all used randomized methods to conduct the surveys, which might compensate for the study’s shortcomings. Second, the survey questionnaires used in the many and various consulted studies were not completely unified in style. However, overall, the survey content of the various questionnaires was relatively consistent. In addition, we searched the literature that was focused on the cognition of the importance of and demand for medical statistics and performed a data extraction and a comprehensive comparative analysis, thus strengthening our description of the research problem. Finally, the reform of medical statistics education and teaching in China proposed in this paper must be tested in practice and requires additional extensive and in-depth investigation.

## Conclusions

Based on the demand of medical students and medical staff members for medical statistics education in China, these individuals are aware that their competency in terms of practical applications is insufficient. However, their recognition of the importance of medical statistics is increasing, and the demand for training in its practical applications is expanding. We believe that medical statistics education and its reforms should be based on the practical demands of medical staff and medical students to improve their capacity to apply medical statistics to their clinical and research practices.

## Supporting Information

S1 Fig Meta-Analysis of Cognition of Basic Statistical Methods (Descriptive statistics and t-test) in Medical Statistics. (I-squared and *P* were the heterogeneity test criteria; ◇pooled cognition rate;—■—, cognition rate and 95% confidence interval).
(TIF)

S2 Fig Meta-Analysis of Cognition of Basic Statistical Methods (One-way ANOVA and Chi-squared test) in Medical Statistics. (I-squared and *P* were the heterogeneity test criteria; ◇pooled cognition rate;—■—, cognition rate and 95% confidence interval).
(TIF)

S3 Fig Meta-Analysis of Cognition of Basic Statistical Methods (Nonparametric test and Correlation and Regression) in Medical Statistics. (I-squared and *P* were the heterogeneity test criteria; ◇pooled cognition rate;—■—, cognition rate and 95% confidence interval).
(TIF)

S4 Fig Meta-Analysis of Cognition of Basic Statistical Methods (Statistical Graph & Table and Statistical Design) in Medical Statistics. (I-squared and *P* were the heterogeneity test criteria; ◇pooled cognition rate;—■—, cognition rate and 95% confidence interval).
(TIF)

S5 Fig Meta-Analysis of Cognition of Advanced Statistical Methods (Multiple ANOVA, Analysis of Covariance, Multiple Linear Regression and Logistic Regression) in Medical Statistics. (I-squared and *P* were the heterogeneity test criteria; ◇pooled cognition rate;—■—, cognition rate and 95% confidence interval).
(TIF)

S6 Fig Meta-Analysis of Cognition of Advanced Statistical Methods (Survival Analysis, Discriminant Analysis, Clustering Analysis and PCA & FA) in Medical Statistics. (I-squared and *P* were the heterogeneity test criteria; ◇pooled cognition rate;—■—, cognition rate and 95% confidence interval).
(TIF)

S7 Fig Meta-Analysis of Demand for Basic Statistical Methods (Descriptive Statistics, t-test, One-way ANOVA, Chi-squared Test, Nonparametric Test, Correlation and Regression) in Medical Statistics. (I-squared and *P* were the heterogeneity test criteria; ◇pooled demand rate;—■—, demand rate and 95% confidence interval).
(TIF)

S8 Fig Meta-Analysis of Demand for Statistical Methods (Statistical Graphs & Tables, Statistical Design, Multiple ANOVA, Analysis of Covariance, Multiple Linear Regression, and Logistic Regression) in Medical Statistics. (I-squared and *P* were the heterogeneity test criteria; ◇pooled demand rate;—■—, demand rate and 95% confidence interval).
(TIF)

S9 Fig Meta-Analysis of Demand for Advanced Statistical Methods (Survival Analysis, Discriminant Analysis, Clustering Analysis, and PCA & FA) in Medical Statistics. (I-squared and *P* were the heterogeneity test criteria; ◇pooled demand rate;—■—, demand rate and 95% confidence interval).
(TIF)

S10 Fig The Usage Rates for Statistical Software (SPSS and SAS). (I-squared and *P* were the heterogeneity test criteria; ◇pooled usage rate;—■—, usage rate and 95% confidence interval).
(TIF)

S1 File PRISMA 2009 Checklist. (DOC)

S1 Table Quality Assessment Results. (DOC)

## References

[1] Freeman JV, Collie S, Staniforth D, Smith KJ. Innovations in curriculum design: A multi-disciplinary approach to teaching statistics to undergraduate medical students. BMC Medical Education 2008; 8: 28 10.1186/1472-6920-8-28 18452599 PMC2397402

[2] Bland M. An Introduction to Medical Statistics, edn 3 Oxford: Oxford University Press; 2001.

[3] Cox DR. Some challenges for medical statistics. European Journal of Epidemiology 2005; 20: 5–9. 15756898 10.1007/s10654-004-6997-2

[4] Miles S, Price GM, Swift L, Shepstone L, Leinster SJL. Statistics teaching in medical school: Opinions of practicing doctors. BMC Medical Education 2010; 10:75 10.1186/1472-6920-10-75 21050444 PMC2987935

[5] Kanter MH, Taylor JR. Accuracy of statistical methods in transfusion: a review of articles from July/ August 1992 through June 1993. Transfusion, 1994; 34: 697–701. 8073487 10.1046/j.1537-2995.1994.34894353466.x

[6] Kuritani T. Key points in medical statistics-erroneous application of statistical methods IV. Nihon Seirigaku Zasshi 1996; 58: 377–384. 9036184

[7] West CP, Ficalora RD. Clinician Attitudes toward Biostatistics. Mayo Clin Proc 2007; 82: 939–943. 17673062 10.4065/82.8.939

[8] Monge E. Most frequent mistakes in medical statistics. Rev Gastroenterol Peru 2008; 28: 11–12. 18418451

[9] He J, Jin Z, Yu D. Statistical reporting in Chinese biomedical journals. Lancet 2009; 2002 373: 2091–2093. 10.1016/S0140-6736(09)60867-9 19446326

[10] Astin J, Jenkins T, Moore L. Medical students’ perspective on the teaching of medical statistics in the undergraduate medical curriculum. Statist Med 2002; 21: 1003–1006. 10.1002/sim.1132 11921009

[11] Clayden AD. Who should teach medical statistics, when, how and where should it be taught? Stat Med 1990; 9: 1031–1037. 2244076 10.1002/sim.4780090906

[12] Sayed-Hassan RM, Bashour HN, Koudsi AY. Patient attitudes towards medical students at Damascus University teaching hospitals. BMC Medical Education, 2012; 12: 13 10.1186/1472-6920-12-13 22439893 PMC3317872

[13] Rostom A, Dube C, Cranney A, Saloojee N, Sy R, Garritty C, et al. Celiac Disease. Rockville (MD): Agency for Healthcare Research and Quality (US); 2004 Sep. (Evidence Reports/Technology Assessments, No.104.) Appendix D. Quality Assessment Forms. http://www.ncbi.nlm.nih.gov/books/NBK35156.

[14] Fang YG, Wu YN, Jian RS, Chen LY, Jiang X. Survey on the knowledge of and demand for medical statistics and countermeasures for continuing education for nursing staff. Chinese Journal of Health Statistics 2011; 28: 559–561. (In Chinese)

[15] Hu Dongmei, Liu Qigui. Survey on the demand for medical statistics in graduates of medical colleges. Chinese Journal of Medical Education Research 2011; 10:761–762. (In Chinese)

[16] Li Lixia, Gao Yanhui, Zhou Shudong, Zhang Min, Ye Xiaohua, Xu Ying, et al Research on cognition of the practical teaching mode of health statistics in preventive medicine students. Journal of Mathematical Medicine 2013; 26:66–68. (In Chinese)

[17] Ma Haiyan, Liu Tingjie, Xu Liangwen. Survey on the demand for medical statistics by health management professionals. Health Vocational Education 2011; 29:124–125. (In Chinese)

[18] Ma Xiuqiang, Meng Hong, Lu Jian, He Jia. Cross-sectional study on the cognition and demand for medical statistics for PhD students. Chinese Journal of Health Statistics 2009; 26:531–532. (In Chinese)

[19] Meng H, Zhang LM, He J, Lu j, Ma XQ. Research on the demand of doctoral graduate students for medical statistics teaching. Journal of Shanxi Medical University (Preclinical Medical Education Edition) 2009; 11: 549–551. (In Chinese)

[20] Qi Guangzi, Ren Meixuan, Huang Gaoming. Survey on the mastery and demand of medical statistics in different types of medical graduates. Chinese Journal of Medical Education Research 2011; 10:1459–1463. (In Chinese)

[21] Sun Yalin, Zhang Luoman, Meng Hong, He Jia, Lu Jian, Ma Xiuqiang. Investigation of the need for two-phased teaching of medical statistics for a seven-year program in undergraduate clinical medicine. Northwest Medical Education 2009; 17:542–545. (In Chinese)

[22] Tang J, Hu PC. Current situational analysis of the cognition of clinicians regarding medical statistics and educational needs. Modern Medicine Health 2011; 27: 622–623. (In Chinese)

[23] Wang Jing, Ye Dongqing, Xie Yipeng, Zhang Chengye, Fan Yinguang, Gao Rong. Investigation and analysis of the present status of SPSS software in health statistics teaching. Northwest Medical Education 2007; 15:75–76. (In Chinese)

[24] Wu Juan, Li Guochun, Dong Ju, Zhang Junfang. Teaching methods discussion about medical statistics for nursing undergraduates. Chinese Journal of Modern Nursing 2011; 17: 4356–4359. (In Chinese)

[25] Yu Canqing, He Jing, Hu Yonghua. (2011) Survey on the application of attitudes and behaviors in medical statistics by public health graduates. Chinese Journal of Medical Education 2011; 31:604–606. (In Chinese)

[26] Zhang Huayan. Investigation and analysis of medical statistics in research design and academic papers at primary comprehensive hospitals. Chin J Prim Med Pharm 2011; 14: 2004–2005. (In Chinese)

[27] Zhang YQ, Yi D, Wu YZ, Liu L, Zhao ZW. Investigation of the need for two-phased teaching of medical statistics in an eight-year program for medical students. China Higher Medical Education 2012; 26: 133. (In Chinese)

[28] Zhao Yanfang, Ma Xiuqiang, Meng Hong, Lu Jian, Meng Hong, Guo Xiaojing, et al Cross-sectional study on the cognition of medical statistics in graduates. Chinese Journal of Health Statistics 2013; 30: 112–113. (In Chinese)

[29] Zhu Yan, Zhu Junmin, Hu Jin, et al Survey on the teaching effect and demand of medical statistics. Journal of Guiyang Medical College 2013; 38:329–331. (In Chinese)

[30] Wu Y, Zhang L, Liu L, Zhang Y, Liu X, Yi D. Attitudes of medical graduate and undergraduate students toward the learning and application of medical statistics. Journal of Biological Education Jun 2014; 10.1080/00219266.2014.923487(online)

[31] Wang Runhua, Yi Jing, Zhong Xiaoni. New ideas about the teaching of medical statistics for medical postgraduate students. Researches in Medical Education 2010; 9: 511–513. (In Chinese)

[32] Chen Ya, Deng Qin, Yao Jia. Analyses of the necessity and measures of teaching reform for medical statistics. Chinese Journal of Health Statistics 2009; 26:317–322. (In Chinese)

[33] Vandenbroeck P, Wouters L, Molenberghs G, Van Gestel J, Bijnens L. Teaching statistical thinking to life scientists: a case-based approach. J Biopharm Stat 2006; 16: 61–75. 16440837 10.1080/10543400500406520

[34] Rockhold FW. Strategic use of statistical thinking in drug development. Statist Med 2009; 19: 3211–3217. 10.1002/1097-0258(20001215)19:23<3211::aid-sim622>3.0.co;2-f 11113955

[35] Lange N. Statistical thinking in functional and structural magnetic resonance neuroimaging. Statist Med 1999; 18: 2401–2407. 10.1002/(sici)1097-0258(19990915/30)18:17/18<2401::aid-sim264>3.0.co;2-z 10474148

[36] Morita S. A role of statistical thinking in clinical anesthesia practice. Masui 1992; 41: 1966–1976. 1479666

[37] Altekar M, Homon CA, Kashem MA, Mason SW, Nelson RM, Patnaude LA, Yingling J, Taylor PB. Assay optimization: a statistical design of experiments approach. Clin Lab Med 2007; 27:139–54. 17416307 10.1016/j.cll.2007.01.001

[38] Parsons NR, Price CL, Hiskens R, Achten J, Costa ML. An evaluation of the quality of statistical design and analysis of published medical research: results from a systematic survey of general orthopaedic journals. BMC Med Res Methodol 2012; 12: 60 10.1186/1471-2288-12-60 22533688 PMC3476984

[39] Hess KR. Statistical design considerations in animal studies published recently in cancer research. Cancer Res 2011; 71: 625 10.1158/0008-5472.CAN-10-3296 21239476

[40] Virkler K, Lednev IK. Blood species identification for forensic purposes using Raman spectroscopy combined with advanced statistical analysis. Anal Chem 2009; 81: 7773–7777. 10.1021/ac901350a 19670872

[41] Gou L, Yu J, Yang Z. Appropriate application of statistical analysis methods in medical science and technology articles. Zhongguo Xiu Fu Chong Jian Wai Ke Za Zhi 2005; 19: 925–927. (In Chinese) 16334245

[42] Labenz J, Kunz CU. The ABC's of medical statistics. Reading and understanding clinical trials. Anaesthesist 2011; 60: 79–89. 10.1007/s00101-010-1840-5 21264653

[43] Pandolfi M, Carreras G. Recent trends in medical statistics: their relevance to evidence-based medicine and to complementary alternative medicine. Eur J Ophthalmol 2011; 21: 1–4. 20853256

[44] Branca M, Morosini P, Severi P, Erzen M, Di Benedetto C, Syrjänen K. New statistical software for intralaboratory and interlaboratory quality control in clinical cytology. Validation in a simulation study on clinical samples. Acta Cytol 2005; 49: 398–404. 16124169 10.1159/000326173

[45] Garvey MT, O'Sullivan M, Blake M. (2000) Multidisciplinary case-based learning for undergraduate students. Eur J Dent Educ 2000; 4: 165–168. 11168482 10.1034/j.1600-0579.2000.040404.x

[46] Patterson JS. (2006) Increased student self-confidence in clinical reasoning skills associated with case-based learning (CBL). J Vet Med Educ 2006; 33: 426–431. 17035220 10.3138/jvme.33.3.426

[47] Bland JM. Teaching statistics to medical students using problem-based learning: the Australian experience. BMC Medical Education 2004; 4: 31 15588318 10.1186/1472-6920-4-31 PMC539273

[48] Prince KJ, Van Eijs PW, Boshuizen HP, Van der Vleuten CP, Scherpbier AJ. General competencies of problem-based learning (PBL) and non-PBL graduates. Med Educ 2005; 39: 394–401. 15813762 10.1111/j.1365-2929.2005.02107.x

[49] Zhang YQ, Wang WC, Liu L, Wu YZ, Yi D. Practice of problem-based learning in the teaching of medical statistics to eight-year program medical students. Journal of Shanxi Medical University (Preclinical Medical Education Edition) 2010; 12:977–980. (In Chinese)

