# Correction: Cognition of and Demand for Education and Teaching in Medical Statistics in China: A Systematic Review and Meta-Analysis

**DOI:** 10.1371/journal.pone.0145517

**Published:** 2015-12-16

**Authors:** Yazhou Wu, Liang Zhou, Gaoming Li, Dali Yi, Xiaojiao Wu, Xiaoyu Liu, Yanqi Zhang, Ling Liu, Dong Yi

The image for S6 Fig is incorrect. Please view the correct S6 Fig below.

## Supporting Information

S6 Fig Meta-Analysis of Cognition of Advanced Statistical Methods (Survival Analysis, Discriminant Analysis, Clustering Analysis and PCA & FA) in Medical Statistics. (I-squared and *P* were the heterogeneity test criteria; ◇pooled cognition rate;—■—, cognition rate and 95% confidence interval).
(TIF)
