# Supplementary figures and images for: Cognition of and Demand for Education and Teaching in Medical Statistics in China: A Systematic Review and Meta-Analysis
Source: PLoS One. 2015 Jun 8;10(6):e0128721. doi: 10.1371/journal.pone.0128721 (PMC4459963; doi:10.1371/journal.pone.0128721)

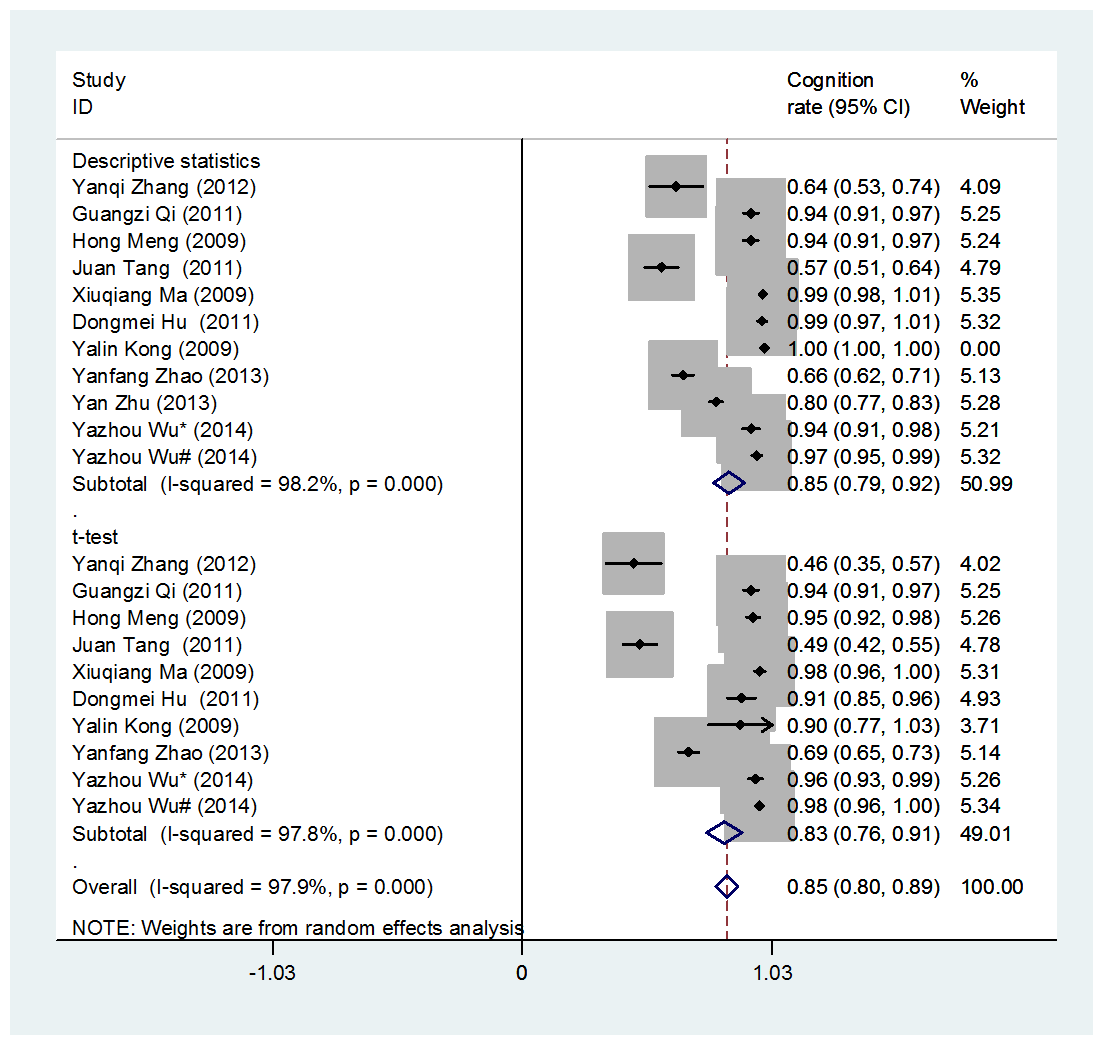

Supplement: S1 Fig — (I-squared and P were the heterogeneity test criteria; ◇pooled cognition rate;—■—, cognition rate and 95% confidence interval). (TIF) [file pone.0128721.s001.tif]

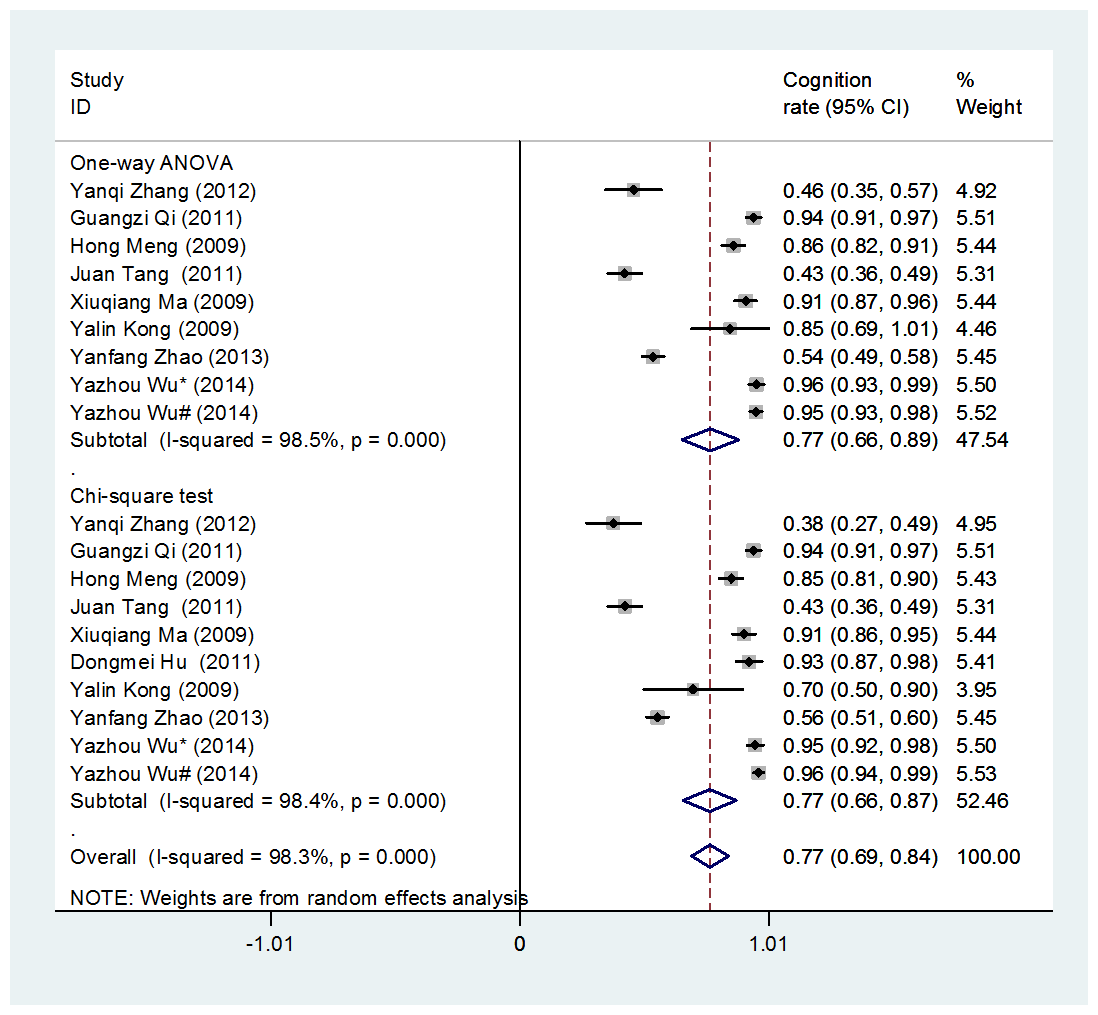

Supplement: S2 Fig — (I-squared and P were the heterogeneity test criteria; ◇pooled cognition rate;—■—, cognition rate and 95% confidence interval). (TIF) [file pone.0128721.s002.tif]

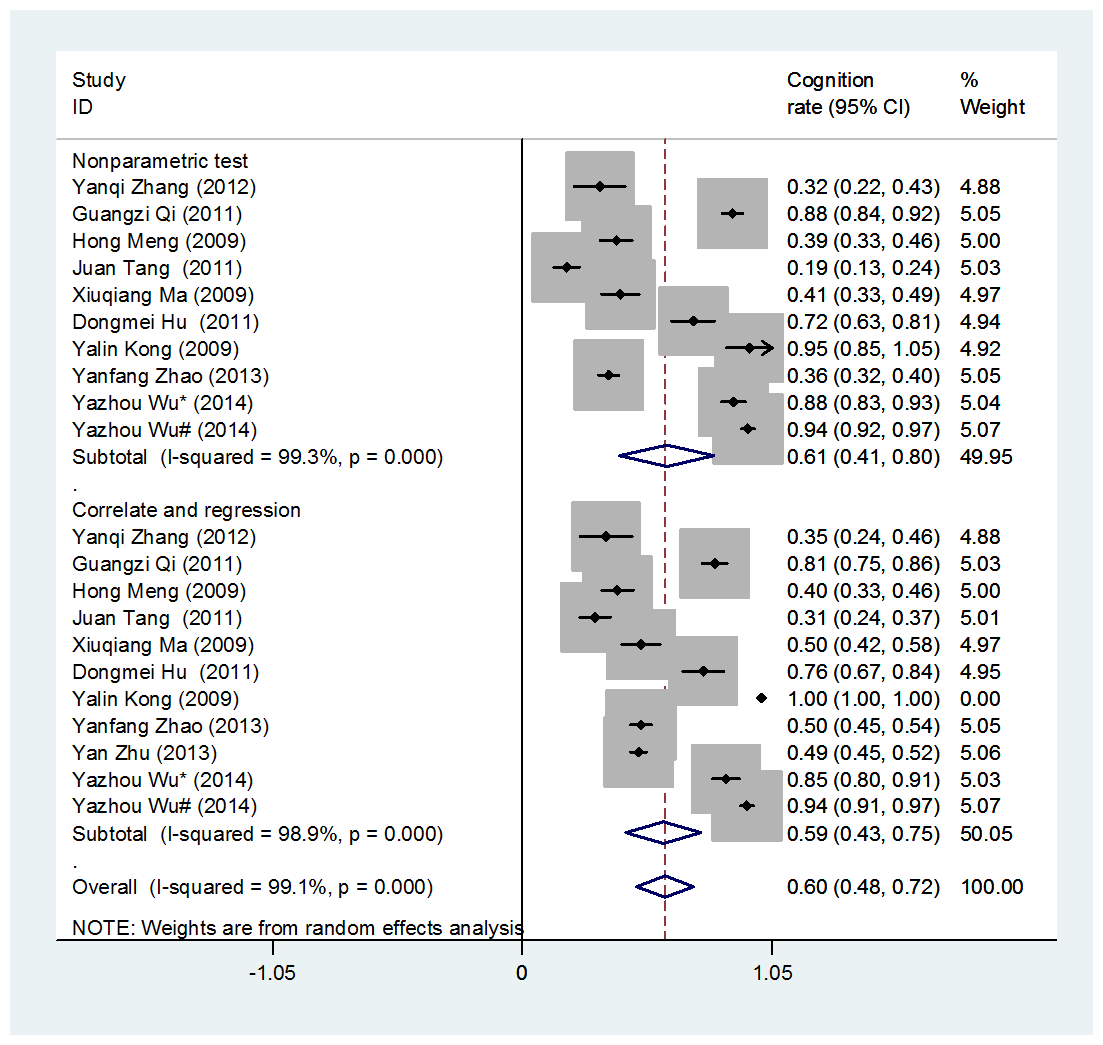

Supplement: S3 Fig — (I-squared and P were the heterogeneity test criteria; ◇pooled cognition rate;—■—, cognition rate and 95% confidence interval). (TIF) [file pone.0128721.s003.tif]

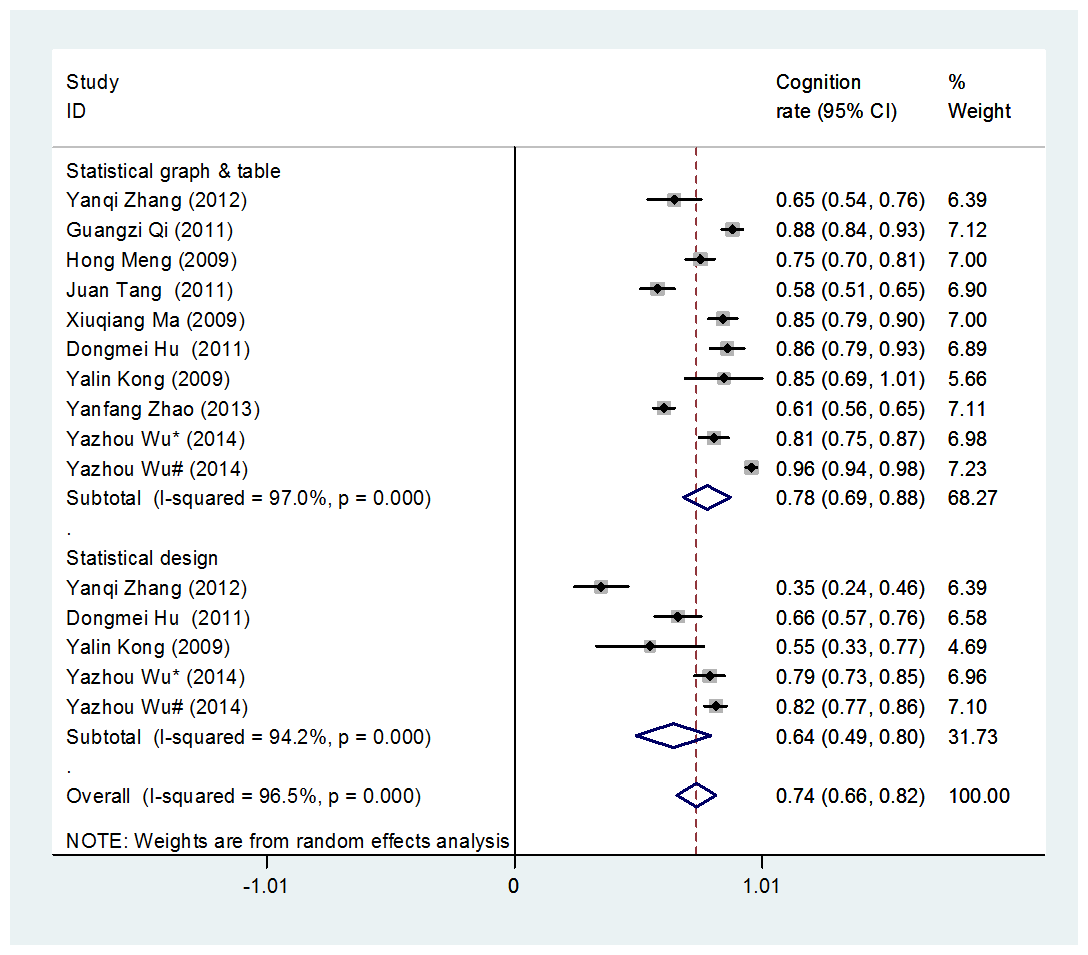

Supplement: S4 Fig — (I-squared and P were the heterogeneity test criteria; ◇pooled cognition rate;—■—, cognition rate and 95% confidence interval). (TIF) [file pone.0128721.s004.tif]

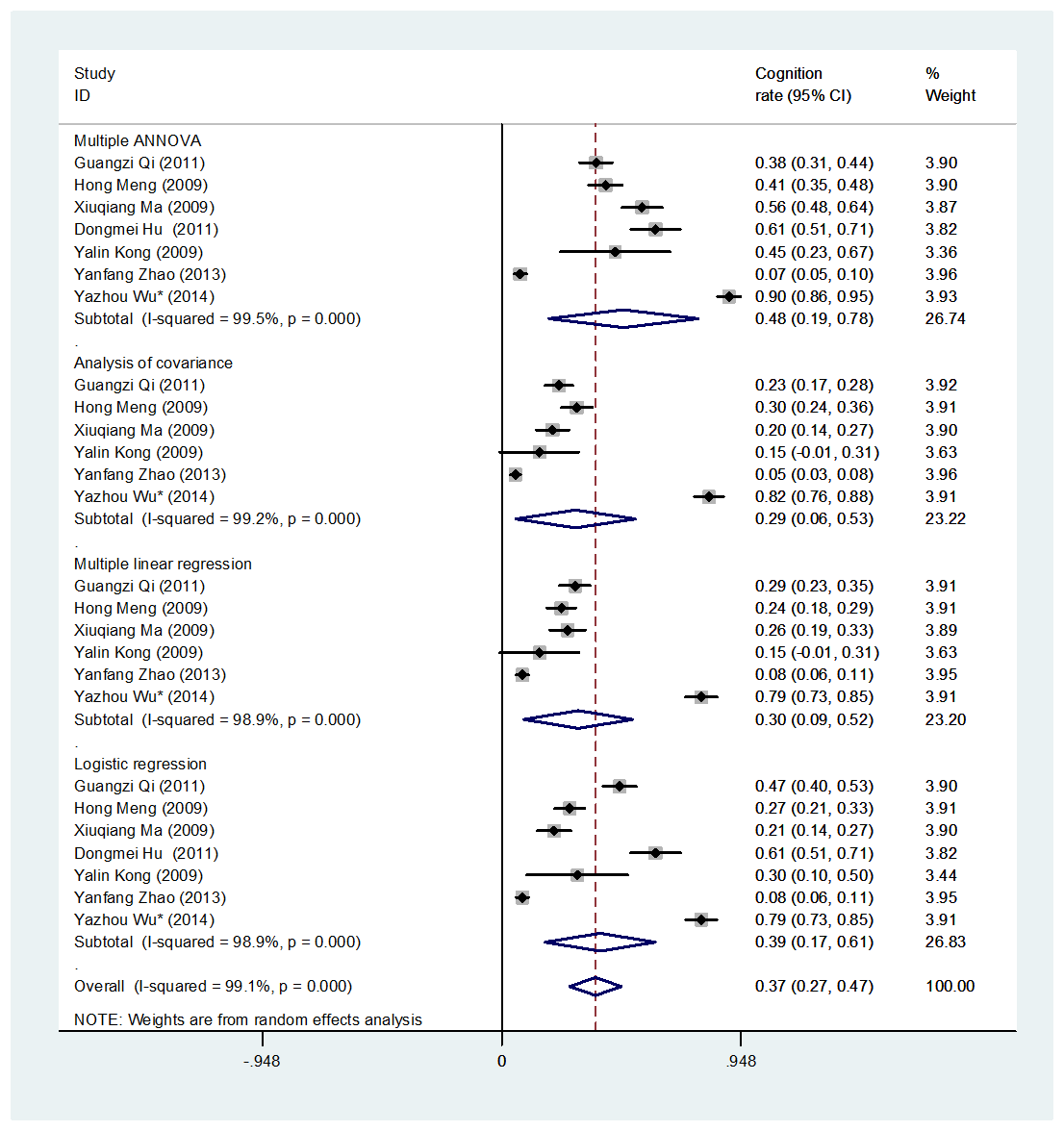

Supplement: S5 Fig — (I-squared and P were the heterogeneity test criteria; ◇pooled cognition rate;—■—, cognition rate and 95% confidence interval). (TIF) [file pone.0128721.s005.tif]

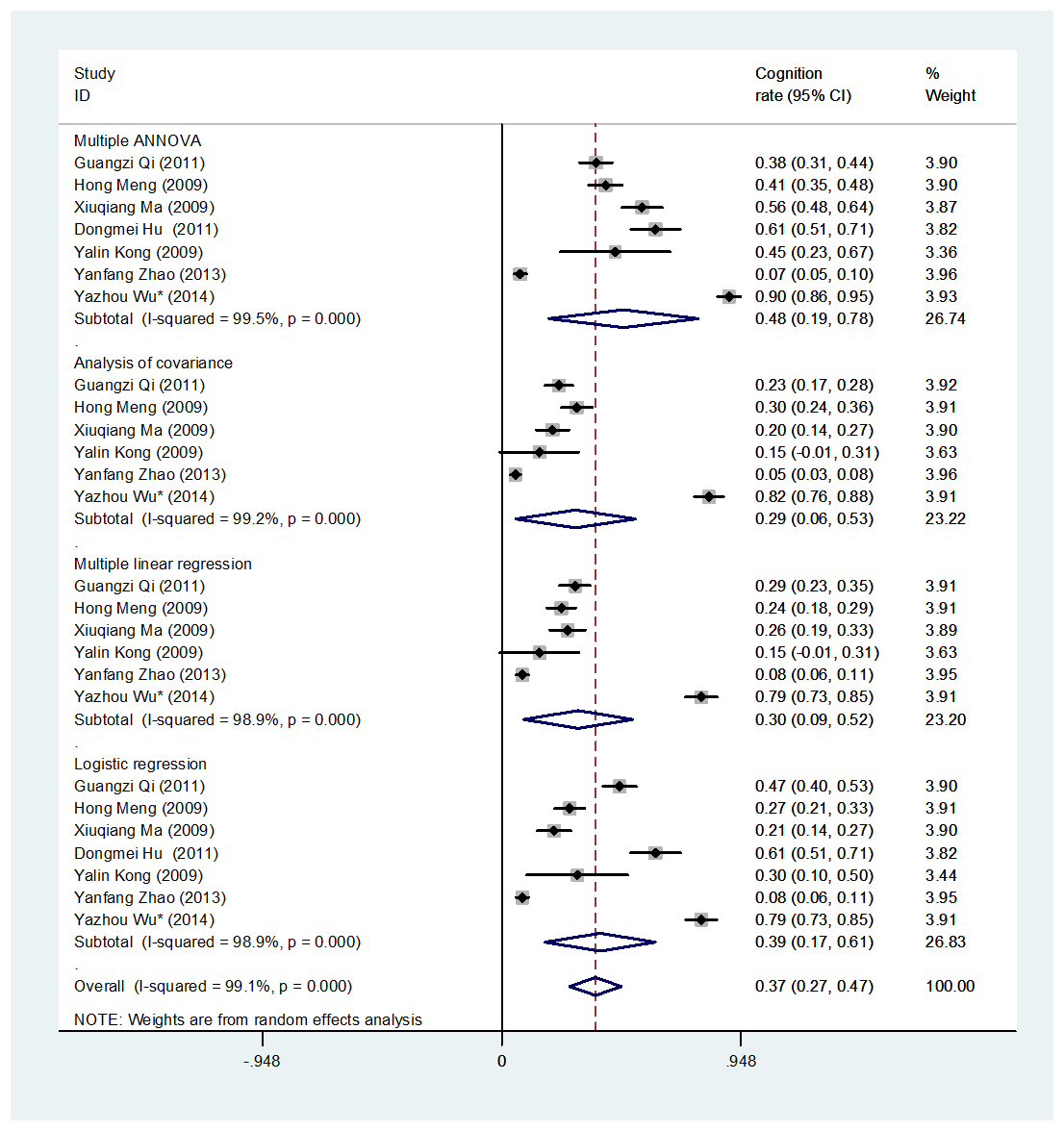

Supplement: S6 Fig — (I-squared and P were the heterogeneity test criteria; ◇pooled cognition rate;—■—, cognition rate and 95% confidence interval). (TIF) [file pone.0128721.s006.tif]

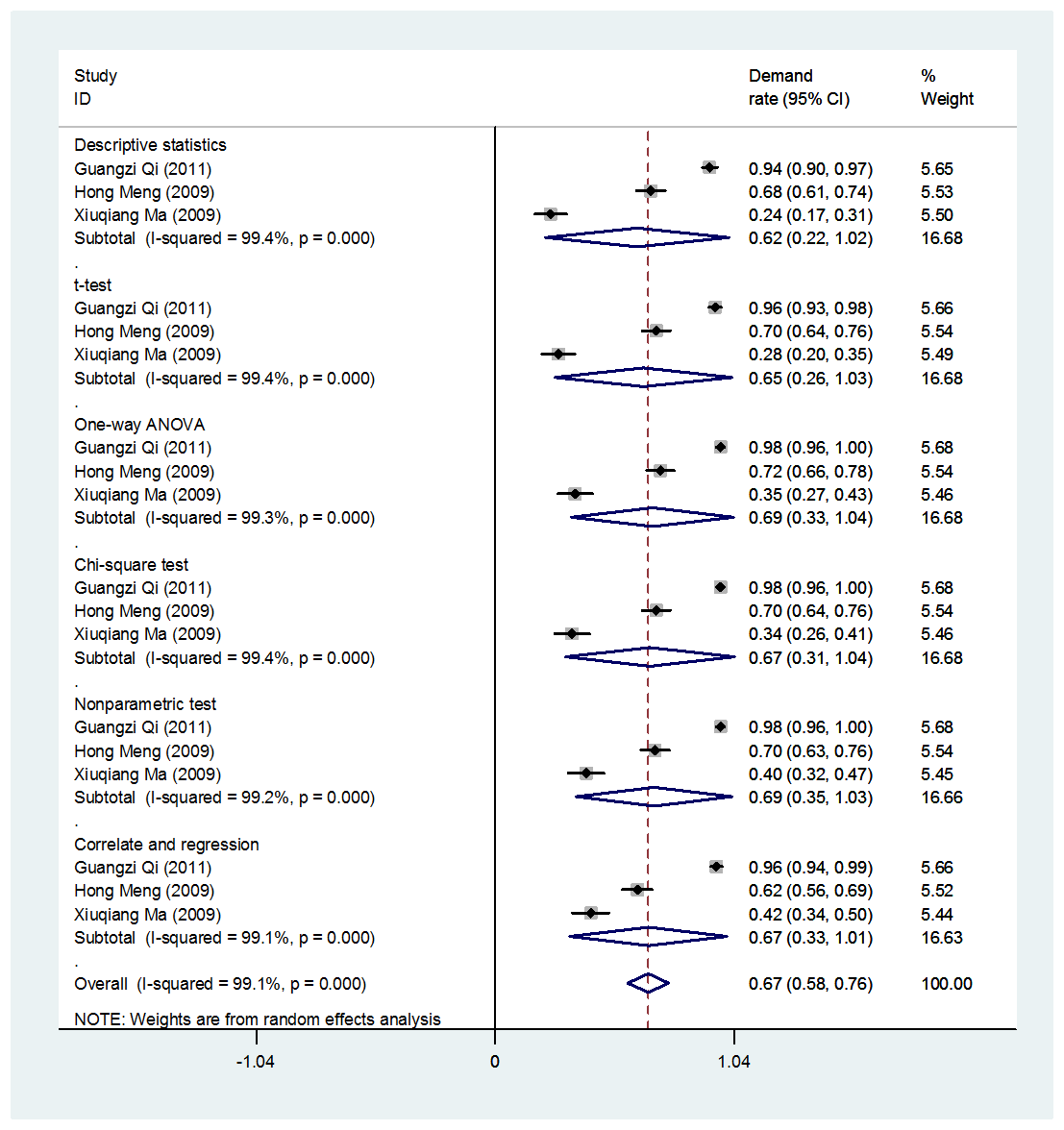

Supplement: S7 Fig — (I-squared and P were the heterogeneity test criteria; ◇pooled demand rate;—■—, demand rate and 95% confidence interval). (TIF) [file pone.0128721.s007.tif]

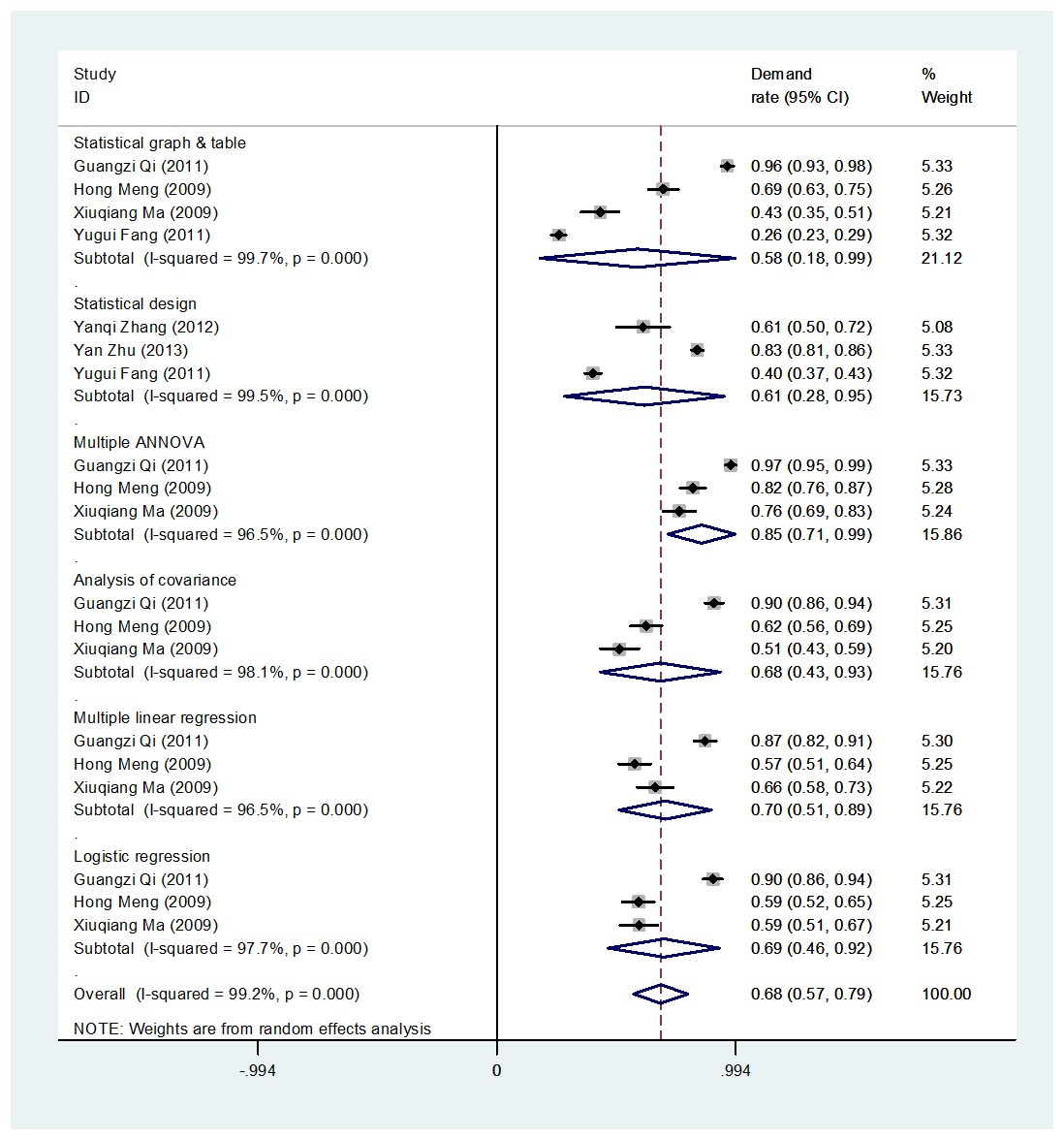

Supplement: S8 Fig — (I-squared and P were the heterogeneity test criteria; ◇pooled demand rate;—■—, demand rate and 95% confidence interval). (TIF) [file pone.0128721.s008.tif]

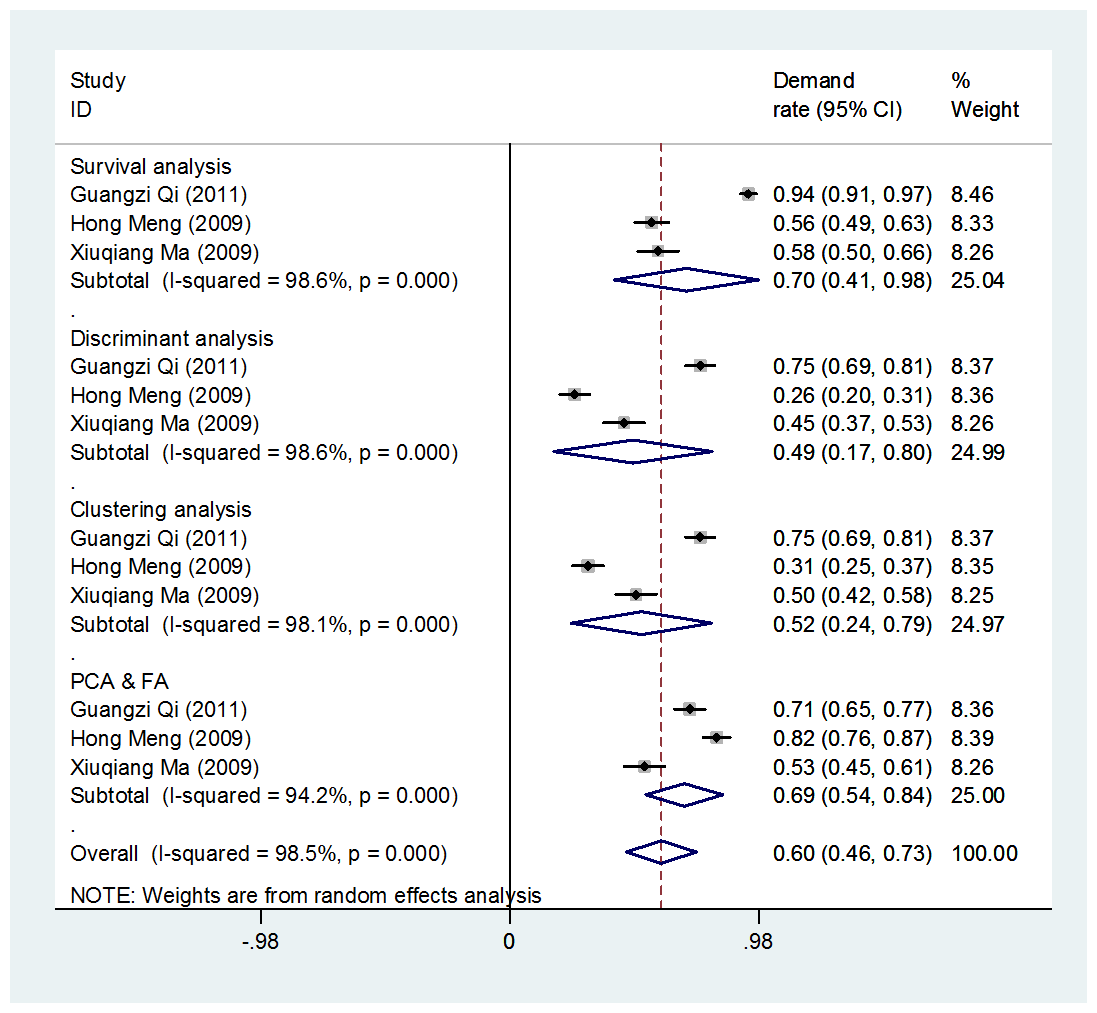

Supplement: S9 Fig — (I-squared and P were the heterogeneity test criteria; ◇pooled demand rate;—■—, demand rate and 95% confidence interval). (TIF) [file pone.0128721.s009.tif]

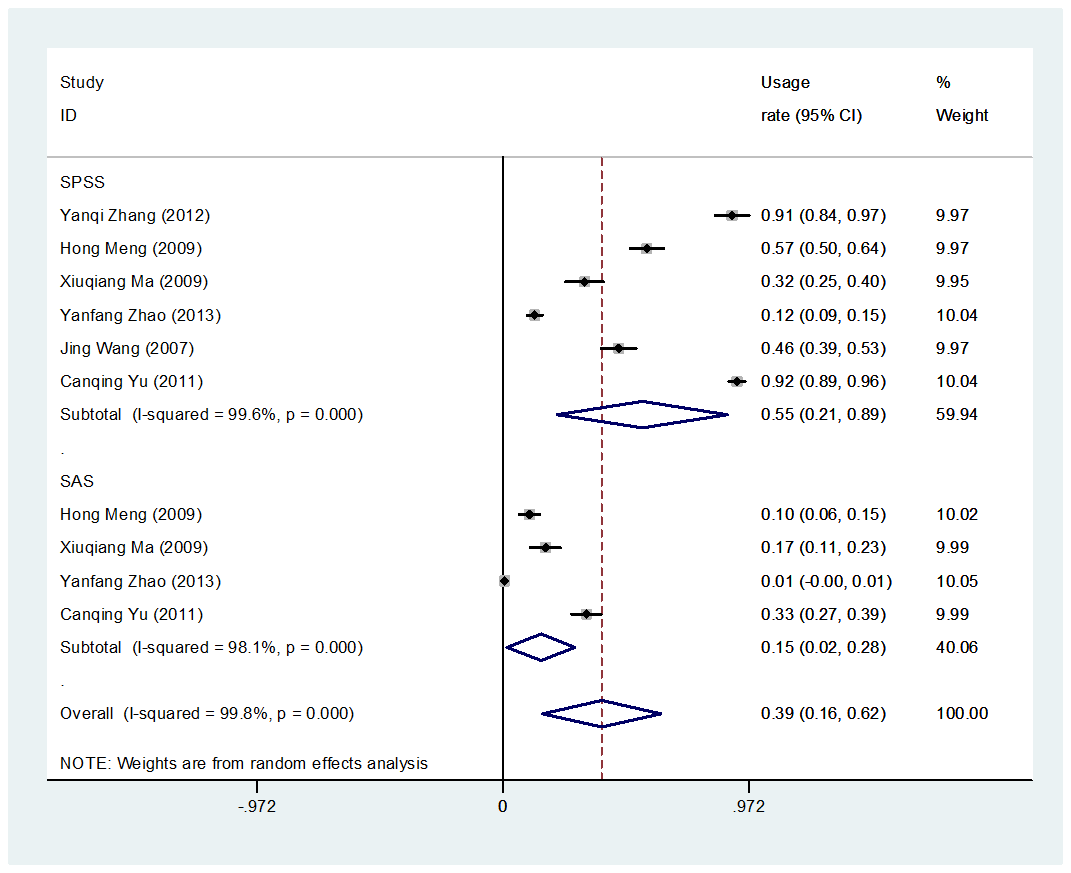

Supplement: S10 Fig — (I-squared and P were the heterogeneity test criteria; ◇pooled usage rate;—■—, usage rate and 95% confidence interval). (TIF) [file pone.0128721.s010.tif]
