# Supplementary material for: Cognition of and Demand for Education and Teaching in Medical Statistics in China: A Systematic Review and Meta-Analysis
Source: PLoS One. 2015 Jun 8;10(6):e0128721. doi: 10.1371/journal.pone.0128721 (PMC4459963; doi:10.1371/journal.pone.0128721)
Supplement: S1 File — (DOC) [file pone.0128721.s011.doc]

| **Section/topic** | **#** | **Checklist item** | **Reported on page #** |
| --- | --- | --- | --- |
| **TITLE** | | |  |
| Title | 1 | Cognition of and Demand for Education and Teaching of Medical Statistics in China: a Systematic Review and Meta-Analysis. | 1 |
| **ABSTRACT** | | |  |
| Structured summary | 2 | **Background:** Although much literature focuses on the teaching and applications of medical statistics in China, very few studies comprehensively evaluate the cognition of and demand for medical statistics; the results of various studies differ and are not sufficiently comprehensive and systematic. **Objectives:** The present investigation aimed to evaluate the cognition of and demand for medical statistics comprehensively intended for undergraduates, graduates, and medical staff in China.  **Methods:** We performed a comprehensive database search related to the cognition of and demand for medical statistics from January 2007 to Jul 2014 and conducted a meta-analysis of non-controlled studies with sub-group analysis for undergraduates, graduates, and medical staff.  **Results:** There is much difference in the cognition of theory in medical statistics among undergraduates (73.5%), graduates (60.7%), and medical staff (39.6%). The demand for theory in medical statistics is very high among graduates (94.6%), undergraduates (86.1%), and medical staff (88.3%). Regarding specific statistical methods, the cognition of basic statistical methods is higher than that of advanced statistical methods; the demand for certain advanced statistical methods, including (but not limited to) multiple ANOVA, multiple linear regression, and logistic regression, is higher than that for basic statistical methods. The usage rates of SPSS and SAS are only 55% and 15%, respectively.  **Conclusion:** The overall statistical competence of undergraduates, graduates, and medical staff is insufficient, and their ability to practically apply their statistical knowledge is limited, which constitutes an unsatisfactory state of affairs for medical statistics education. Because the demand for skills in this area is presently increasing, the need to reform medical statistics education in China has become urgent. | 2 |
| **INTRODUCTION** | | |  |
| Rationale | 3 | At present, although many publications focus on the teaching and applications of medical statistics in China, very few studies comprehensively evaluate the cognition of and demand for medical statistics; even among such documents, the results of various studies differ and are not sufficiently comprehensive and systematic. | 3 |
| Objectives | 4 | Therefore, we performed a comprehensive search of the relevant literature regarding the cognition of and demand for medical statistics. Using comprehensive comparisons of the cognition of and demand for medical statistics among undergraduates, graduates, and medical staff, we conducted a comprehensive review and evaluated the present situation of the cognition of and demand for medical statistics. Finally, we analyzed and summarized the existing problems in medical statistics and proposed a targeted improvement strategy, which provides a reference and basis for innovative education and teaching reform of medical statistics courses and improves the ability of clinical researchers and medical students to use analytical statistical knowledge to solve practical problems in medicine. | 3 |
| **METHODS** | | |  |
| Protocol and registration | 5 | N/A | N/A |
| Eligibility criteria | 6 | The literature inclusion criteria were as follows:  ①The included studies were all investigations.  ②The subjects of this study included 3 types of people at the medical university and hospital: undergraduates, graduates, and medical staff.  ③The study investigated the cognition of and demand for medical statistics methods and the conditions for using statistics software among undergraduates, graduates, and medical staff in China.  ④End indicators included the cognition level, the demand level, and the usage level for medical statistics methods or related software.  The literature exclusion criteria were as follows:  ①Literature with non-exploitable data or a vague concept of cognition and demand in the study;  ②Review literature;  ③Literature with serious data omissions. | 3-4 |
| Information sources | 7 | We used ‘‘teaching methods’’ as the title word and ‘‘cognition’’, “demand” or “need”, ‘‘medical statistics’’ or “health statistics”, and “Biometry” as the keywords to search the following databases, during the period from January 2007 to December 2013. | 4 |
| Search | 8 | PubMed, Medline, Chinese Biomedical Literature Database, China Doctoral Dissertations Full-text Database, Chinese Scientific and Technological Journals Database, Traditional Chinese Medicine Database, China Doctoral Dissertations Full-text Database, China Master Dissertations Full-text Database, and CENTRAL from the Cochrane Library. | 4 |
| Study selection | 9 | ①Literature with non-exploitable data or a vague concept of cognition and demand in the study;  ②Review literature;  ③Literature with serious data omissions. | 4 |
| Data collection process | 10 | Three investigators participated in the data extraction of all publications included in the study. Information, including the first author, publication year, total number of cases included in the study, study object, and endpoint evaluation indicators, was extracted. One investigator (Li GM) first performed the data extraction, and the second investigator (Zhou L) subsequently re-examined the publication and verified the results. Differences were discussed with the third investigator (Wu YZ), and consensus was reached by discussion. | 4-5 |
| Data items | 11 | In terms of data extraction and quantification, the cognition rate, the demand rate, and the usage rate were used as end indicators. Competency in medical statistics was divided into cognition and non-cognition, with the so-called cognition being a core competency of understanding or grasping statistical knowledge and applying that knowledge to solve actual problems. The needs related to medical statistics are divided into demand and non-demand. The so-called demand is due to the lack of medical statistics knowledge and the lack of actual problem-solving ability; therefore, we must further improve learning and training in medical statistics theory or software. | 5 |
| Risk of bias in individual studies | 12 | The included observational studies were subjected to a comprehensive quality assessment using the Newcastle-Ottawa Scale (cross-sectional/Prevalence Study) as a guide [13]. This quality evaluation was performed in a blinded manner by two researchers (Yi D & Liu L) from the second research team, who assigned quality scores to the included studies. The studies that were given different quality scores by the two researchers were referred to a third researcher (Zhang YQ) from this research team for evaluation, and a final quality score was obtained. | 4 |
| Summary measures | 13 | The measurement data were expressed as$\bar{x}\pm s$, and the enumeration data were expressed as a percentage or constituent ratio and rate, and the enumeration data were evaluated using the pooled rate and 95% CI based on the levels of cognition and demand for theory courses and software in medical statistics. | 5 |
| Synthesis of results | 14 | Subgroup meta-analysis for undergraduates, graduates, and medical staff was also used in this study. If the heterogeneity across the studies was within the acceptable range (I^2^<50%), a fixed effects model was used to combine the studies. Otherwise, a random effects model was used. P<0.05 indicated a statistically significant difference. | 5 |

Page 1 of 2

| **Section/topic** | **#** | **Checklist item** | **Reported on page #** |
| --- | --- | --- | --- |
| Risk of bias across studies | 15 | In the result of Newcastle-Ottawa Scale (cross-sectional/Prevalence Study), there are 66.67% (12/18) of the including study above 4 scores in the item of NOS, 22.22% (4/18) of the including study above 7 scores in the item of NOS, and 11.11% (2/18) of the including study above 10 scores in the item of NOS. (Table 1S of the appendix) | 6 |
| Additional analyses | 16 | Table 2 and table 3. | 19 |
| **RESULTS** | | |  |
| Study selection | 17 | In total, 174 research articles on the cognition of and demand for medical statistics were identified by searching electronic databases and other sources. Based on the inclusion and exclusion criteria, 98 articles, including duplicated publications, articles with mismatched titles, and articles with mismatched subjects, were excluded. The remaining 34 articles were reviewed thoroughly, and the following 20 studies were excluded: 8 articles with non-exploitable results, 6 review articles, 3 articles with missing data, and 3 articles with vague concepts of cognition and demand. Thus, 14 studies were included in this study for the systematic review [14-30]. Figure 1 displays the flowchart of the included studies. | 5 |
| Study characteristics | 18 | Table 1 presents the basic demographic characteristics of the undergraduates, graduates, and medical staff in the included studies. The undergraduates included the combined BS/MD Program (7 or 8 years) students. The graduates also included PhD students. The medical staff also included clinicians, nursing personnel, and health service management personnel. In China's education system, undergraduates are college students who are pursuing a bachelor's degree. Graduates are students who are pursuing a master's degree after obtaining a bachelor's degree. PhD students are pursuing a doctoral degree after obtaining a master's degree. Clinicians and nursing personnel are health care workers who are employed at a hospital. | 5-6 |
| Risk of bias within studies | 19 | In the result of Newcastle-Ottawa Scale (cross-sectional/Prevalence Study), there are 66.67% (12/18) of the including study above 4 scores in the item of NOS, 22.22% (4/18) of the including study above 7 scores in the item of NOS, and 11.11% (2/18) of the including study above 10 scores in the item of NOS. (Table 1S of the appendix) | 6 |
| Results of individual studies | 20 | Table 2 and table 3. | 19 |
| Synthesis of results | 21 | **Overall Cognition of and Demand for Medical Statistics Competency Issues**  Table 2 shows the overall cognition of and demand for theory and software, with respect to medical statistics competency issues among undergraduates, graduates, and medical staff. Figures 2, 3, 4, and 5 show the merged results of the meta-analysis of the overall cognition of and demand for medical statistics theory and software. The results from Table 2 and the figures also reveal the following: The cognition rates for medical statistics theory in undergraduates, graduates, and medical staffs were 73.5%, 60.7%, and 39.6%, respectively; the cognition rates for statistics software were 63.3%, 80.8%, and 11.5%, respectively. The demand rates for medical statistics theory among undergraduates, graduates, and medical staff were 86.1%, 94.6%, and 88.3%, respectively; the demand rates for statistics software were 64.7%, 85.7%, and 66.7%, respectively.  **Cognition of and Demand for Statistical Methods and Software**  Table 3 presents the meta-analysis of the statistical methods and software with respect to medical statistics competency issues (Meta-analysis results of each method are shown in Figure S1-S10 of the appendix). Figure 6 shows the relative rates of change in the cognition of and demand for statistical methods.  **Cognition of Statistical Methods:** Among the basic statistical methods, the highest cognition was for descriptive statistics (85.4%), followed by the t-test (83.4%), and one-way ANOVA (77%). The lowest cognition was for correlation and regression (59%) and the nonparametric test (60.5%), and the cognition for experimental and survey design was only 64.5%. Among the advanced statistical methods, the highest cognition was for multiple ANOVA (48.3%), followed by logistic regression (39%) and survival analysis (32.6%); the lowest cognition was for PCA & FA (14.2%).  **Demand for Statistical Methods:** Among the basic statistical methods, the highest demand was for the nonparametric test (69.3%), followed by one-way ANOVA (68.5%) and the chi-square test (67.4%); the lowest demand was for statistical graphs and tables (58.5%), and the demand for experimental and survey design reached up to 61.4%. Among the advanced statistical methods, the highest demand was for multiple ANOVA (85.1%), followed by multiple linear regression (70%) and survival analysis (69.6%); the lowest demand, which was for discriminant analysis, reached up to 48.5%. | 5-6 |
| Risk of bias across studies | 22 | In the result of Newcastle-Ottawa Scale (cross-sectional/Prevalence Study), there are 66.67% (12/18) of the including study above 4 scores in the item of NOS, 22.22% (4/18) of the including study above 7 scores in the item of NOS, and 11.11% (2/18) of the including study above 10 scores in the item of NOS. (Table 1S of the appendix) | 6 |
| Additional analysis | 23 | Table 2 and table 3. | 19 |
| **DISCUSSION** | | |  |
| Summary of evidence | 24 | Problems Discovered;  Adopting Strategies;  First, attention should be paid to the cultivation of statistical thinking;  Second, content regarding statistical design and advanced statistical methods should be improved;  Again, the ability to use statistical software and to apply statistics to practical and clinical applications should be enhanced;  Finally, we should attempt to make the teaching methods flexible and the teaching styles diverse. | 7-9 |
| Limitations | 25 | First, even though the sampled research subjects were relatively representative (clinicians, Ph.D. students, graduates, combined BS/MD program (7 or 8 years) students, and undergraduates), these studies were completed at a single institute. Further, some of the surveyed samples were not sufficiently large or representative of the actual population. However, the students and personnel surveyed are native to many and various regions of China, which is a strength of this study.  The distribution was relatively broad, and the assorted institutes (centers) all used randomized methods to conduct surveys of the subjects, which might compensate for the study’s shortcomings. Second, the survey questionnaires from the many and various studies were not completely unified in style; however, overall, the survey content of the various questionnaires was relatively consistent. Further, we searched the literature focusing on the cognition of the importance of and demand for medical statistics; we performed a data extraction and a comprehensive comparative analysis, thus strengthening our description of this problem. Finally, the reform of medical statistics education and teaching in China proposed herein still must be tested in practice and requires further extensive and in-depth investigations. | 9 |
| Conclusions | 26 | Judging from medical students’ and medical staff members’ demand for medical statistics education in China, they are aware that their competency in terms of practical applications is insufficient. However, their recognition of the importance of medical statistics is increasing, and the demand for training in its practical applications is expanding. We believe that medical statistics education and its reforms should be based on the practical demands of medical staff and medical students to improve their capacity to apply medical statistics to their clinical and research practices. | 9-10 |
| **FUNDING** | | |  |
| Funding | 27 | This work was partially supported by the National Natural Science Foundation of China (No.81273178, No.81172773), Higher Education Scientific Research Subject of the Higher Education Institute from 2013 to 2014 in Chongqing (No.CQGJ13C652), and Research Projects of Postgraduate Education and Teaching Reform in Chongqing, China (No.yjg123101). The funders had no role in study design, data collection and analysis, decision to publish, or preparation of the manuscript. | 10 |

*From:*  Moher D, Liberati A, Tetzlaff J, Altman DG, The PRISMA Group (2009). Preferred Reporting Items for Systematic Reviews and Meta-Analyses: The PRISMA Statement. PLoS Med 6(6): e1000097. doi:10.1371/journal.pmed1000097

For more information, visit: **www.prisma-statement.org**.

Page 2 of 2
