# Supplementary material for: Cognition of and Demand for Education and Teaching in Medical Statistics in China: A Systematic Review and Meta-Analysis
Source: PLoS One. 2015 Jun 8;10(6):e0128721. doi: 10.1371/journal.pone.0128721 (PMC4459963; doi:10.1371/journal.pone.0128721)
Supplement: S1 Table — (DOC) [file pone.0128721.s012.doc]

Table S1. The Result of Quality Assessment

| Study | ⑴ | ⑵ | ⑶ | ⑷ | ⑸ | ⑹ | ⑺ | ⑻ | ⑼ | ⑽ | ⑾ | ⑿ |
| --- | --- | --- | --- | --- | --- | --- | --- | --- | --- | --- | --- | --- |
| Jing Wang^14^ | Yes | No | Yes | Yes | Unclear | Yes | Unclear | Unclear | No | Yes | Unclear | 5 |
| Yalin Sun^15^ | Yes | No | Unclear | Yes | Unclear | Yes | Unclear | No | No | Yes | Yes | 4 |
| Xiuqiang Ma^16^ | Yes | No | Unclear | Yes | Unclear | Yes | Unclear | Unclear | No | Yes | Unclear | 4 |
| Hong Meng^17^ | Yes | No | Unclear | Yes | Yes | Yes | Yes | No | Yes | Yes | Yes | 8 |
| Yugui Fang^18^ | Yes | No | Yes | Yes | Yes | Yes | Unclear | No | Yes | Yes | Yes | 8 |
| Canqing Yu^19^ | Yes | No | No | Yes | Unclear | Yes | Unclear | No | No | Yes | Unclear | 4 |
| Guangzi Qi^20^ | Unclear | No | Unclear | Yes | Yes | Yes | Unclear | No | No | Yes | Unclear | 4 |
| Juan Tang^21^ | Yes | No | Yes | Yes | Unclear | Yes | No | Unclear | No | Yes | Unclear | 5 |
| Dongmei Hu^22^ | Yes | No | No | Yes | Yes | Yes | Yes | Unclear | No | Yes | Yes | 7 |
| Huayan Zhang^23^ | Unclear | No | Yes | Yes | Yes | Yes | Unclear | No | Yes | Yes | No | 6 |
| Haiyan Ma^24^ | Yes | No | Unclear | Yes | Yes | Yes | No | No | No | Yes | Yes | 6 |
| Juan Wu^25^ | Yes | No | No | Yes | Unclear | Yes | No | No | No | Yes | Unclear | 4 |
| Yanqi Zhang^26^ | Unclear | No | Yes | Yes | Unclear | Yes | Yes | Unclear | Yes | Yes | Unclear | 6 |
| Yanfang Zhao^27^ | Yes | No | Unclear | Yes | Yes | Yes | Unclear | Unclear | No | Yes | Yes | 6 |
| Yan Zhu^28^ | Yes | No | Unclear | Yes | Yes | Yes | Unclear | No | Yes | Yes | Yes | 7 |
| LiXia Li^29^ | Yes | No | Yes | Yes | Yes | Yes | No | No | No | Yes | No | 6 |
| Yazhou Wu^30^ | Yes | No | Yes | Yes | Yes | Yes | Yes | Yes | Yes | Yes | Yes | 10 |

⑴Define the source of information (survey, record review);

⑵List inclusion and exclusion criteria for exposed and unexposed subjects (cases and controls) or refer to previous publications;

⑶Indicate time period used for identifying patients;

⑷ Indicate whether or not subjects were consecutive if not population-based;

⑸Indicate if evaluators of subjective components of study were masked to other aspects of the status of the participants;

⑹Describe any assessments undertaken for quality assurance purposes (e.g., test/retest of primary outcome measurements);

⑺Explain any patient exclusions from analysis;

⑻Describe how confounding was assessed and/or controlled;

⑼ If applicable, explain how missing data were handled in the analysis;

⑽Summarize patient response rates and completeness of data collection;

⑾Clarify what follow-up, if any, was expected and the percentage of patients for which incomplete data or follow-up was obtained

⑿Total scores

NOTE: Yes, 1 score; No or Unclear, 0 score. High quality: 8-11; middle quality:4-7; low quality: 1-3.
