# Supplementary figures and images for: Correction: Cognition of and Demand for Education and Teaching in Medical Statistics in China: A Systematic Review and Meta-Analysis
Source: PLoS One. 2015 Dec 16;10(12):e0145517. doi: 10.1371/journal.pone.0145517 (PMC4681532; doi:10.1371/journal.pone.0145517)

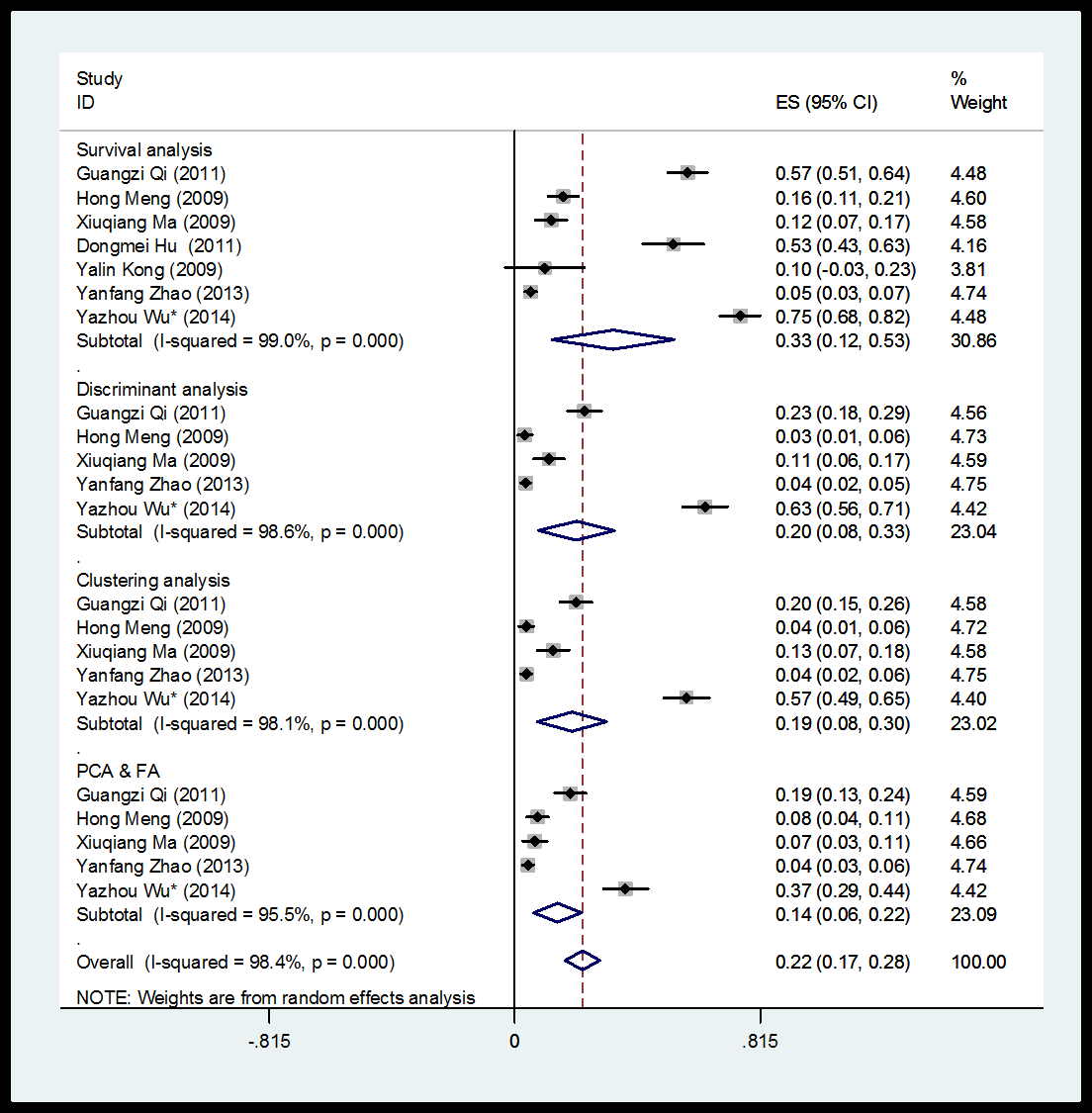

Supplement: S6 Fig — (I-squared and P were the heterogeneity test criteria; ◇pooled cognition rate;—■—, cognition rate and 95% confidence interval). (TIF) [file pone.0145517.s001.tif]
